# Natural Bioactive Compounds in the Management of Type 2 Diabetes and Metabolic (Dysfunction)-Associated Steatotic Liver Disease

**DOI:** 10.3390/ph18020279

**Published:** 2025-02-19

**Authors:** Daniela Ciobârcă, Adriana Florinela Cătoi, Laura Gavrilaș, Roxana Banc, Doina Miere, Lorena Filip

**Affiliations:** 1 Department 2, Faculty of Nursing and Health Sciences, “Iuliu Hatieganu” University of Medicine and Pharmacy, 23 Gheorghe Marinescu Street, 400337 Cluj-Napoca, Romania; muresan.daniela@umfcluj.ro (D.C.); laura.gavrilas@umfcluj.ro (L.G.); 2 Department of Pathophysiology, Faculty of Medicine, “Iuliu Hatieganu” University of Medicine and Pharmacy, 2-4 Victor Babes Street, 400012 Cluj-Napoca, Romania; 3 Department of Bromatology, Hygiene, Nutrition, Faculty of Pharmacy, “Iuliu Hatieganu” University of Medicine and Pharmacy, 6 Louis Pasteur Street, 400349 Cluj-Napoca, Romania; roxana.banc@umfcluj.ro (R.B.); dmiere@umfcluj.ro (D.M.); lfilip@umfcluj.ro (L.F.); 4 Academy of Romanian Scientists (AOSR), 3 Ilfov Street, 050044 Bucharest, Romania

**Keywords:** type 2 diabetes, metabolic (dysfunction)-associated steatotic liver disease, non-alcoholic fatty liver disease, gut dysbiosis, natural bioactive compounds, berberine, curcumin, soluble fibers, omega-3 fatty acids

## Abstract

Type 2 diabetes (T2D) and metabolic (dysfunction)-associated steatotic liver disease (MASLD) affect a growing number of individuals worldwide. T2D and MASLD often coexist and substantially elevate the risk of adverse hepatic and cardiovascular clinical outcomes. Several common pathogenetic mechanisms are responsible for T2D and MASLD onset and progression, including insulin resistance, oxidative stress, and low-grade inflammation, among others. The latter can also be induced by gut microbiota and its derived metabolites. Natural bioactive compounds (NBCs) have been reported for their therapeutic potential in both T2D and MASLD. A large amount of evidence obtained from clinical trials suggests that compounds like berberine, curcumin, soluble fibers, and omega-3 fatty acids exhibit significant hypoglycemic, hypolipidemic, and hepatoprotective activity in humans and may be employed as adjunct therapy in T2D and MASLD management. In this review, the role of the most studied NBCs in the management of T2D and MASLD is discussed, emphasizing recent clinical evidence supporting these compounds’ efficacy and safety. Also, prebiotics that act against metabolic dysfunction by modulating gut microbiota are evaluated.

## 1. Introduction

Type 2 diabetes (T2D), metabolic (dysfunction)-associated steatotic liver disease (MASLD), and obesity are cardiometabolic diseases (CMDs) that pose a substantial burden to global health due to their dramatically increased prevalence over the past decades [1,2]. T2D, which is closely linked to the obesity epidemic, remains a major public health issue and significantly enhances cardiovascular morbidity and mortality [3]. Globally, 415 million individuals have diabetes, of which >90% are diagnosed with T2D [4]. Individuals with T2D are at high risk for developing both micro- and macrovascular complications in the context of hyperglycemia and other components of metabolic syndrome (MS) [3]. Nearly two-thirds of deaths among T2D patients are related to cardiovascular diseases (CVDs), of which around 40% result from ischemic heart disease [5].

MASLD, previously known as non-alcoholic fatty liver disease (NAFLD), is a form of MS that manifests in the liver [6] and is strongly associated with both obesity and T2D. MASLD prevalence is rising alongside global trends in obesity and T2D [7], currently affecting 38% of adults globally [2] and between 70 and 80% of patients with T2D [8]. The spectrum of MASLD extends from simple steatosis (accumulation of lipids in hepatocytes) to steatohepatitis (steatosis, inflammation), cirrhosis, and hepatocellular carcinoma [6]. However, beyond the risk of hepatic complications, the main cause of mortality among patients with MASLD are CVDs [9]. The latter are responsible for approximately one-third of all deaths in patients with MASLD [10].

The relationship between T2D and MASLD is complex and bidirectional, in which one condition can precede or worsen the other [7]. This link is related to the roles of abdominal obesity and insulin resistance (IR), which are underlying traits of MS, in the pathogenesis of both T2D and MASLD [9]. In the last decade, gut microbiota has also been recognized as a potentially driving factor in the onset or progression of these diseases [11,12]. To emphasize its association with T2D, NAFLD has been recently redefined as MASLD, requiring the presence of at least one of five cardiometabolic risk factors (Table 1). Also, non-alcoholic steatohepatitis (NASH) has been reclassified as metabolic (dysfunction)-associated steatohepatitis (MASH) [6]. Since the MASLD/MASH terminology is applicable to populations initially diagnosed with NAFLD/NASH [13], this review adopts the updated MASLD nomenclature. Nevertheless, when referencing earlier clinical studies discussed in Section 4 and Section 5, the original terminology will be retained.

Patients with T2D face more than double the risk of developing cirrhosis and liver cancer compared to the general population, such that international guidelines now recommend screening for MASLD-related liver fibrosis among patients with T2D [15]. The co-occurrence of MASLD in patients with T2D doubles the risk for the onset and development of CVDs compared to patients without T2D [16]. Owing to the tight interaction between T2D and MASLD, therapeutic agents targeting hyperglycemia, insulin sensitivity, and other cardiometabolic risk factors might contribute to the effective management of hepatic steatosis [17].

In recent years, various natural bioactive compounds (NBCs) and their derivatives have emerged as adjuvant alternatives, complementing a healthy lifestyle and conventional treatment, to help alleviate CMDs. The increasing research interest and growing popularity of NBCs with metabolic health benefits are important for several reasons. Firstly, natural entities, characterized by tremendous diversity and structural complexity, have been and continue to be a valuable source for drug discovery. Modern pharmacological therapies for the treatment of T2D are highly effective in improving glycemic control, but their use may be associated with certain adverse effects [18]. Moreover, conventional lipid-lowering treatments exhibit variable effectiveness in improving the blood lipid profile, and often residual cardiovascular risk persists [19]. For example, the prevalence of lipid abnormalities among T2D patients on statin-lowering therapy was found to be up to 50%, rendering them susceptible to increased residual cardiovascular risk [20]. On the other hand, statin intolerance, which manifests as statin-associated muscle symptoms, often leads to low treatment adherence, augmenting the risk for cardiovascular events [21]. Therefore, exploring NBCs from natural sources and their clinical applications could lead to the development of innovative medications with fewer side effects and improved efficacy [18]. Secondly, long-term adherence to lifestyle intervention, the first-line therapeutic approach for metabolic abnormalities, is often poor [22] and insufficient to reduce risk factors. Hence, multi-drug therapy is often required, which is also associated with reduced compliance, in addition to drug-drug interactions and adverse effects [23]. Thirdly, despite our extensive knowledge about MASLD pathogenesis, no pharmacological therapies have been approved so far [7]. For all these reasons, natural adjuvant therapy may be a promising strategy to help achieve treatment targets, improve outcomes, delay disease progression, and ultimately reduce cardiovascular risk.

The current review aims to describe the efficacy and safety profile of selective NBCs with hypoglycemic effects that also exhibit hypolipidemic and hepatoprotective properties, summarizing the scientific evidence from recent human clinical trials that supports the beneficial role of these compounds in improving glucose and lipid metabolism. We also address the role of prebiotics in managing T2D and MASLD via their impact on gut microbiota. 

## 2. Methodology

A comprehensive literature search was conducted on Pubmed, Science Direct, and Google Scholar, using various keywords and medical subject headings (MeSH), such as T2D, fatty liver diseases—hepatic steatosis, NAFLD, NASH, MASLD, and MASH—as well as selected NBCs like berberine, curcumin (turmeric), resveratrol, anthocyanins, catechins, green tea, phenolic compounds, artichoke, carotenoids, lycopene, hesperidin, silymarin, cinnamon, fenugreek, soluble fibers/prebiotics, and omega-3 fatty acids.

The selection of articles was performed according to the following criteria: (i) articles written in English published in the last 25 years; (ii) studies focusing on adult human subjects, particularly randomized controlled trials, but also systematic reviews and meta-analyses of such trials, as well as narrative reviews; (iii) articles whose outcomes included glycemic control (fasting blood glucose, postprandial blood glucose, glycosylated hemoglobin, homeostasis model assessment for insulin resistance), blood lipid profile (total cholesterol, plasma triglycerides, LDL cholesterol, HDL cholesterol), liver-related parameters (alanine aminotransferase, aspartate aminotransferase, gamma-glutamyl transpeptidase or alkaline phosphatase, liver steatosis/fibrosis scores), inflammatory/anti-inflammatory markers (C-reactive protein, interleukin-6, interleukin-10, tumor necrosis factor α, adiponectin, etc.), antioxidant status (total antioxidant capacity, malondialdehyde, glutathione peroxidase, superoxide dismutase, etc.), and anthropometric indices (BMI, body weight, body fat percentage, waist circumference, waist-to-hip ratio). In vitro research, studies using animal models, and clinical studies with observational designs were excluded.

## 3. Pathogenetic Mechanisms Leading to T2D and MASLD

**Figure 1:**
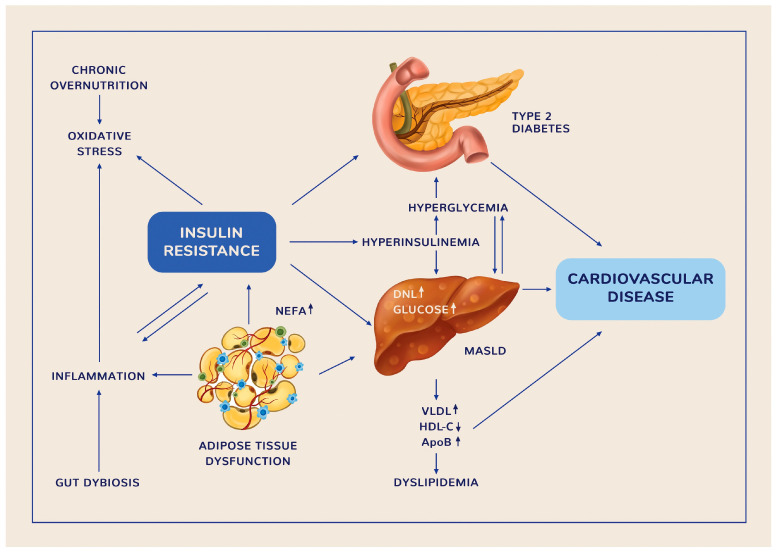
IR as a central mechanism in the pathogenesis of T2D and MASLD [27,28]. ApoB, apolipoprotein B; DNL, de novo lipogenesis; NEFA, non-esterified plasma free fatty acids; HDL-c, high-density lipoproteins; VLDL, very low-density lipoproteins.

CMDs, such as obesity, T2D, or MASLD, encompass a cluster of processes that impair insulin sensitivity, glucose and lipid metabolism, and immune function. CMDs frequently occur concomitantly, appear to share common pathogenetic mechanisms, and are associated with an increased risk of disability and premature death [24]. They also possess a complex genetic [25] and environmental etiology [3]. Although many aspects of these clinical entities are not fully elucidated, it is believed that IR, inflammation, and oxidative stress are the most common pathogenetic mechanisms underlying their onset and progression [26] (Figure 1).

### 3.1. Insulin Resistance

IR is a clinical condition in which insulin produces a lower-than-expected biological response [29] to suppress hepatic glucose production, stimulate glucose disposal in skeletal muscle, inhibit lipolysis, and promote glycogen synthesis [3]. The IR state requires increased insulin secretion to compensate, leading to high levels of fasting plasma insulinemia. Chronic overnutrition in the context of IR causes hyperinsulinemia, which further exacerbates IR, leading to β-cell failure [30] and liver cell injury [31] due to the toxic effects of excessive glucose and lipids (gluco- and lipotoxicity) [30,31].

T2D mainly results from progressively impaired insulin secretion by β-cells in the context of pre-existing liver, skeletal muscle, and adipose tissue IR, often a consequence of obesity. In the liver, IR and insulin deficiency, in the setting of hyperglucagonemia, increased glucagon sensitivity, and the supply of gluconeogenic substrates (fatty acids, glycerol, lactate, and amino acids), stimulate gluconeogenesis, which results in fasting hyperglycemia [3]. Since endogenous glucose production increases in a state of impaired fasting glucose accompanied by hyperinsulinemia, hepatic IR is the main defect promoting hyperglycemia in the early and intermediate stages of T2D [32]. In skeletal muscle, IR affects insulin’s ability to stimulate glucose disposal [33] by altering glucose transporter type 4 (GLUT4) translocation to the cell surface in response to insulin [34]. Because of its major role in postprandial glucose uptake (up to 80%), skeletal muscle is recognized as a key factor in systemic IR [33]. Factors responsible for skeletal muscle IR include defective insulin signaling, altered glucose transport or glucose phosphorylation, and decreased mitochondrial oxidative capacity. IR in adipose tissue accelerates lipolysis, leading to increased non-esterified plasma free fatty acids (NEFA) levels, which further exacerbate IR in the liver and muscle [3].

IR affects not only the liver, muscle, and adipose tissue but also the kidneys, vasculature, and brain. Increased renal glucose reabsorption and an elevation of the renal threshold for glucose also contribute to fasting hyperglycemia. IR in the vascular endothelium causes metabolic stress by altering the vasodilator action of insulin and subsequently decreases the supply of glucose, as well as insulin itself [3]. In the brain, IR is associated with a decrease or absence of regulatory signals that modulate peripheral metabolism, particularly in the postprandial state. Hence, certain peripheral metabolic responses, including endogenous glucose synthesis, cellular glucose uptake, liver energy metabolism, and pancreatic insulin secretion, are compromised in the state of IR [35]. In summary, T2D is caused by multi-organ IR coupled with a progressive decline in insulin secretion [36].

Adipose tissue IR is characterized by an increased NEFA flux to the liver, which promotes intrahepatic fat accumulation. Excessive NEFA can either be oxidized, reassembled into triglycerides (TGs) and stored in hepatocyte lipid droplets, or released as large very low-density lipoproteins (VLDL) into circulation. Overproduction of VLDL-cholesterol leads to atherogenic dyslipidemia, characterized by increased levels of triglycerides and apolipoprotein B particles, as well as decreased concentrations of HDL-cholesterol. Dyslipidemia is frequently associated with the extent of liver fat accumulation (Figure 1) [37]. Hepatic TG assembly is generally coordinated with VLDL production and stored intracellular TGs. Hence, hepatic fat accumulation occurs when the equilibrium between lipid storage and clearance in the liver becomes disrupted [9]. In addition to lipid overflow due to excessive peripheral lipolysis, de novo lipogenesis (DNL) and increased hepatic uptake of chylomicron remnants and intrahepatically generated VLDL also contribute to liver steatosis [9]. In MASLD, insulin fails to suppress gluconeogenesis and glucose output from the liver, yet continues to increase lipid synthesis, leading to hyperglycemia and hypertriglyceridemia. This process is known as selective IR. It is estimated that, in the setting of both hyperglycemia and hyperinsulinemia, DNL accounts for around 38% of liver triglycerides (TGs), compared to 11% in lean subjects [38].

Mitochondrial dysfunction contributes to MASLD pathogenesis due to the role that mitochondria play in gluco- and lipogenesis, as well as fatty acid oxidation. In IR states, particularly obesity or T2D, hepatic mitochondrial oxidative activity is initially increased to adapt to elevated lipid availability. However, the oxidative capacity of the mitochondria in the liver has been shown to decrease over time in individuals with steatosis, leading to oxidative stress, mitochondrial alterations, and worsening of MASLD [35].

The accumulation of TGs in the liver is believed to be a protective mechanism to prevent endoplasmic reticulum stress, reactive oxygen species synthesis, and lipid intermediate formation [35]. While the storage of lipids as TGs in hepatocytes is relatively benign, the accumulation of cholesterol, phosphatidylcholines, diacylglycerol, and certain saturated fatty acids accentuates lipotoxicity, leading to local IR and inflammation. Chronic adipose tissue-related inflammation and lipotoxicity from excessive fat accumulation in the liver trigger stress-activated signaling pathways that lead to liver cell apoptosis. The liver initiates a healing response, including proliferation and fibrosis, which can eventually progress to more severe conditions, such as cirrhosis or cancer [39].

#### Molecular Mechanisms Underlying IR

Insulin elicits its biological effects by binding to its receptor (INSR) and subsequently activating specific proteins, including insulin receptor substrates (IRSs, particularly IRS-1 and IRS-2) and insulin receptor tyrosine kinase. In turn, phosphorylated IRS proteins trigger major intracellular signaling pathways, such as phosphatidylinositol 3-kinase (PI3K) and RAS-mitogen-activated protein kinase (MAPK). PI3K is responsible for the translocation of GLUT4 to the cell membrane, thereby stimulating glucose uptake in skeletal muscle [3]. The MAPK pathway modulates the mitogenic and proliferative effects of insulin [28]. Defective phosphorylation of IRS proteins (e.g., increased serine and decreased tyrosine phosphorylation) promotes IR [3]. In obesity and T2D, the tyrosine phosphorylation of IRS-1 is inhibited, leading to altered IRS-1 signaling and subsequent skeletal muscle IR [40]. Other potential triggering mechanisms of IRS serine phosphorylation include ectopic lipid accumulation, endoplasmic reticulum stress, mitochondrial dysfunction, and inflammation [3].

### 3.2. Inflammation and Oxidative Stress

Adipose tissue produces various anti-inflammatory and proinflammatory molecules, whose abnormal expression is involved in the development of metabolic dysfunction [41]. Obesity is characterized by a proinflammatory state that decreases the plasma concentration of anti-inflammatory mediators, such as adiponectin (APN) [41] and interleukin (IL) 10 [42]. APN stimulates muscle glucose disposal and fatty acid oxidation while promoting the suppression of hepatic gluconeogenesis [41]. Decreased APN levels have been associated with inflammation markers in obesity-related disorders [43].

Since it was discovered that adipocytes secrete inflammatory mediators and that obesity is characterized by an increased number of macrophages in adipose tissue, adipose tissue inflammation has been considered the primary cause of IR [36]. Highly proinflammatory macrophages, together with adipocytes, produce abnormal cytokine synthesis [e.g., tumor necrosis factor-α (TNF-α), IL-1, and IL-6], increased acute-phase reactants, and mediators, as well as activate inflammatory signaling pathways such as JNK and IKKβ, leading to impaired insulin action [44]. JNK modulates the serine phosphorylation of IRS-1, contributing to the TNF-α-related impairment of insulin signaling [45]. IKKB activates the nuclear transcription factor kappa B (NF-kB), which, in turn, augments the expression of proinflammatory molecules. Inflammasomes, multi-protein complexes activated by intracellular nutrients (e.g., glucose, free fatty acids), are also components of the low-grade inflammation process [46]. In addition to impaired insulin signaling, cytokines and proinflammatory mediators produced by adipocytes and macrophages lead to β-cell failure, impaired vascular flow, and endothelial dysfunction by upregulating various inflammatory pathways [47].

Oxidative stress (OS) is a significant upstream event for inflammation, as it promotes macrophage activation, increased cytokine production, and the inflammatory response, leading to IR, T2D [48], and MASLD [49]. Glucotoxicity leads to an increase in ROS in β-cells [32], causing mitochondrial damage, negative effects on insulin secretion, cellular death, and tissular damage [50]. Moreover, chronically elevated levels of glucose and NEFA exert synergistic harmful effects and maximize β-cell toxicity [51]. ROS derived from dysfunctional mitochondria have been associated with NLRP3 inflammasome activation [52], which controls the secretion of proinflammatory cytokines IL-1β and IL-18 [53]. IL-1β is considered responsible for the development of chronic complications in T2D [54].

Excessive fat accumulation in the liver due to hepatic lipid overflow results in lipotoxicity, with subsequent mitochondrial dysfunction, endoplasmic reticulum stress, and ROS formation. The overproduction of ROS causes mitochondrial injury, hepatic cellular death, and lipid oxidation, leading to inflammation and fibrogenesis [49].

### 3.3. Gut Microbiota-Related Inflammation

Beyond adipose tissue and the liver, the gastrointestinal tract may also be a source of inflammation due to its altered microbiota (dysbiosis), causing IR and metabolic dysfunction. Studies in both animal models and humans have shown that T2D [11] and MASLD [12] are associated with gut microbiota (GM) function and composition abnormalities, although conflicting evidence regarding the specific dysbiotic profiles in these conditions has emerged [11,12].

A large body of evidence highlights the role of the gut–liver axis in the pathogenesis and management of both T2D [11] and MASLD [12]. The gut–liver axis is a bidirectional pathway of communication between the intestine and liver [55] that plays a central role in maintaining energy homeostasis [56]. This bidirectional crosstalk is facilitated, on one hand, by the portal vein, which carries gut-derived products to the liver. In addition, immune cells activated by nutrients or gut metabolites enter lymphatic vessels, influencing immune responses in distal organs. On the other hand, the liver responds to the gut by releasing bile acids (BAs) and other metabolites into the biliary tract and systemic circulation. BAs possess antimicrobial activity, controlling unrestricted bacterial overgrowth. This is crucial for maintaining the normal function of the gut–liver axis. Also, BAs regulate multiple metabolic processes by activating nuclear receptors [55].

Various genetic and environmental factors may disrupt the complex interplay among the GM, intestinal barrier, immune system, and liver, leading to CMDs [57]. Gut barrier dysfunction manifests as increased intestinal permeability, which enables the translocation of microbes and their metabolites [endotoxins, particularly lipopolysaccharide (LPS)] to distal organs through the portal circulation, causing inflammation [55] and IR [58]. Gut dysbiosis is also linked to a reduced number of short-chain fatty acid (SCFA)-producing bacteria, the induction of an immune response against LPS, high levels of ethanol-producing bacteria, and subsequent increased ethanol synthesis, as well as the conversion of choline to trimethylamine and abnormal levels and composition of plasma BAs [59]. The overgrowth of ethanol-producing bacteria may accelerate MASLD progression to more severe forms of liver disease [60].

## 4. Natural Bioactive Compounds for T2D and MASLD Treatment

NBCs are small molecules found in various plant parts or foods that can provide health benefits [61] due to their modulatory effects on metabolic pathways (e.g., cholesterol-lowering or anti-inflammatory activity) [62]. To date, numerous NBCs have been discovered, each possessing its own distinct pharmacologic effects and health benefits. As such, NBCs exert powerful antioxidant, anti-inflammatory, and antimicrobial effects [63], which exhibit hypoglycemic, hepatoprotective, hypolipidemic, hypotensive, and cardioprotective properties [64].

NBCs have a wide range of natural sources and possess strikingly diverse chemical structures and biological activities [63]. They are either primary or secondary metabolites of plants or nutritional components [62], such as polyphenols, flavonoids, alkaloids, tannins, carotenoids, etc. These compounds are found in various parts of plants, such as leaves, bark, and roots, or may naturally occur in foods [61]. For example, fruits, vegetables, grains, seeds, nuts, legumes, herbs, and spices are valuable sources of NBCs. Several NBCs, however, originate from animal sources and have been extensively researched for their functional benefits on human health (e.g., omega-3 PUFA) [62]. In addition to fresh foods, NBCs are common ingredients in nutraceuticals and dietary supplements [64].

**Figure 2:**
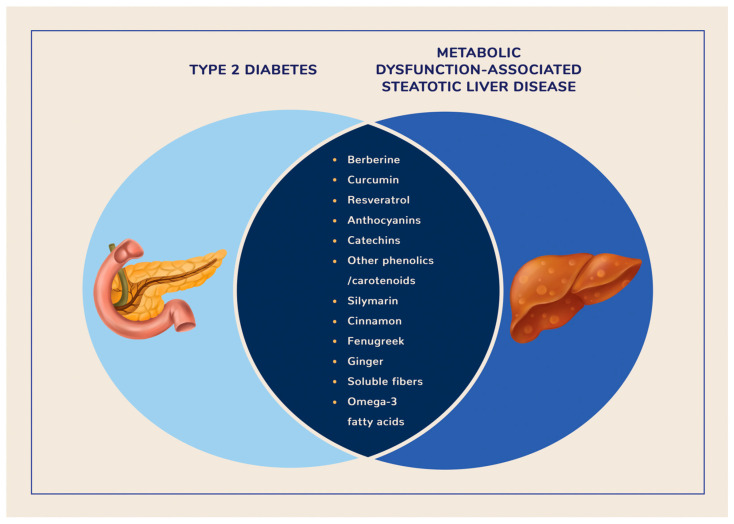
NBCs with potential therapeutic roles in T2D and MASLD.

In this section, we will discuss in detail the clinical evidence supporting the efficacy and safety of specific NBCs with antidiabetic, hypolipidemic, and hepatoprotective activity. The selected NBCs used in the management of both T2D and MASLD are summarized in Figure 2.

### 4.1. Berberine

Berberine (BBR) is an alkaloid extracted from *Rhizoma coptidis* (Huanglian) that has been traditionally used in the management of T2D [65]. BBR’s antidiabetic activity was first reported in 1986, and subsequent in vivo studies confirmed its role in improving glucolipid metabolism [66]. Other reported pharmacological effects of BBR include anti-inflammatory, anti-carcinogenetic [65], antibacterial, anti-platelet aggregation, and cardioprotective properties [67].

*Clinical evidence in T2D.* The glucose- and lipid-lowering effects of BBR were confirmed by multiple randomized clinical trials (RCTs) in patients with T2D (Table 2). Yin et al. (2008) compared the efficacy of BBR monotherapy with metformin monotherapy (1500 mg daily for 3 months) in patients with newly diagnosed T2D (n = 31). Results showed significant improvements in fasting blood glucose (FBG), postprandial blood glucose (PBG), and HbA_1c_ in both groups. BBR’s glucose-lowering effect was compared to that of metformin. In a second study involving 43 patients with poorly controlled T2D, the authors showed that BBR significantly lowered FBG, PBG, and HbA_1c_ levels, along with fasting insulin (FI) plasma concentration and homeostasis model assessment for insulin resistance (HOMA-IR) levels. In addition, BBR supplementation exhibited positive effects on the blood lipid profile in both studies, significantly decreasing plasma total cholesterol (TC) and TGs, as well as TC and LDL-c levels [68].

Zhang et al. (2010) also demonstrated that BBR (1 g/day for 2 months) significantly lowered FBG, HbA_1c_, insulin, and TG levels in patients with T2D (n = 50). BBR exhibited a similar efficacy in improving glycemic control as metformin (n = 26) and rosiglitazone (n = 21) [69].

Another RCT by Gu et al. (2010) analyzed the effects of either BBR or placebo on glucolipid metabolism and indicated that BBR (1 g/day) improved glucose and lipid metabolism parameters in patients with T2D (n = 60). After 3 months, BBR significantly decreased FBG, 2 h PBG, HbA_1c_, TC, TGs, and LDL-c levels compared to placebo. The authors demonstrated that BBR mainly modulated the metabolism of free fatty acids in T2D patients [70].

A 2013 RCT demonstrated that equal doses of BBR (1 g/day) and BBR in combination with silymarin (BBR 1 g/day and SML 210 mg/day) were capable of significantly decreasing FBG, TC, TG, and liver enzyme levels [alanine aminotransferase (ALT) and aspartate transferase (AST]) in patients with T2D and suboptimal glycemic control (n = 63). Moreover, BBR-SLM was proven to be more effective than BBR alone in reducing HbA_1c_ concentration. LDL-c also declined significantly only in the BBR-SLM-supplemented patients, but there were no differences between groups. It was proposed that in combination with SLM, BBR has increased bioavailability, as the former may act as a potential antagonist of P-glycoprotein. The latter mediates the extrusion of BBR from gut cells and promotes its extensive biliary excretion [71].

In clinical practice, most patients on statin-lowering therapy do not reach their LDL-c goal due to statin intolerance [21] or refractory hypercholesterolemia [78], among other factors. Hence, a non-statin hypolipidemic treatment or a lipid-lowering combination therapy is often recommended [21,78]. Recent studies have demonstrated the beneficial effects of proprotein convertase subtilisin/kexin type 9 (PCSK9) inhibitors on lowering LDL-c and cardiovascular risk [79]. Notably, BBR directly inhibits PCSK9 expression [80]. In 2015, a combined therapy of BBR and SLM was administered to patients with T2D and statin intolerance (n = 45). Patients enrolled in the study were divided into three groups receiving a low statin dose (n = 15), ezetimibe (n = 15), or no treatment at all (n = 15). Results showed that BBR and SLM, either as monotherapy or as add-on therapy to statins and ezetimibe, significantly reduced TGs and LDL-c in all patients after 12 months. Also, significant improvements were reported in FBG and HbA_1c_ levels [72].

Dai et al. reported that 300 mg of BBR (n = 36) administered alongside hypoglycemic and hypotensive medication led to a significant decrease in C-reactive protein (CRP) and malondialdehyde (MDA) levels, as well as a significant increase in high molecular weight APN, glutathione peroxidase (GSH-Px), superoxide dismutase (SOD) activity, and total antioxidant capacity (TAC) in T2D patients. However, FBG and HbA_1c_ concentrations did not differ significantly between groups at the end of the study period [73].

More recently, a 2022 systematic review and meta-analysis (SRMA) by Xie et al. reported statistically significant improvements in FBG, HbA_1c_, and 2 h PBG following BBR supplementation in patients with T2D (n = 3048). BBR’s glucose-lowering effect was associated with baseline mean FBG and HbA_1c_ levels. BBR dosages ranged from 0.9 g to 2.4 g/day, while intervention durations varied from 14 days to 6 months [67].

*Clinical evidence in MASLD.* In addition to being a robust oral hypoglycemic and hypolipidemic agent, BBR also exerts positive effects on liver function (Table 2). Yan et al. (2015) performed an RCT involving 155 NAFLD patients who randomly received lifestyle intervention (LSI) alone or in combination with BBR (0.5 g three times daily) or pioglitazone (15 mg/day) for 16 weeks. BBR and LSI significantly lowered hepatic fat content (HFC) compared to LSI alone. Also, significant improvements in blood lipid profiles, HOMA-IR, and body weight (BW) were also observed. BBR was superior to pioglitazone in improving lipid profile levels and BW. Liver enzyme levels declined in all three groups at the end of the study period, but there were no significant differences between the groups [74].

Chang et al. (2016) showed that in patients with NAFLD (n = 80) treated with LSI alone or in combination with BBR (0.5 g three times daily), HFC, TC, and TGs levels were significantly decreased in the intervention group after 16 weeks. However, liver enzyme concentrations, specifically, ALT, AST, and gamma-glutamyl transpeptidase (GGT), did not differ significantly between the BBR and LSI groups at the end of the follow-up period. More significant improvements in anthropometric indices (BMI, BW, WC) were also observed in the intervention group compared to the control group. The lipid-lowering effect of BBR was mediated by the downregulation of circulating ceramides [75].

Harrison et al. (2021) reported that berberine ursodeoxycholate (1 g twice daily for 18 weeks) was more effective than a placebo in decreasing HFC in patients with presumed NASH and T2D (n = 88). Significant decreases in liver enzyme levels (ALT, GGT), HbA_1c_, and BW were also observed in the treatment group compared to the control group [76].

Koperska et al. (2024) demonstrated that, after 12 weeks of treatment, patients with metabolic dysfunction-associated fatty liver disease (MAFLD) (n = 63) receiving BBR (1.5 g/day) showed a statistically significant decrease in ALT, ALT/AST ratio, and TC levels compared to placebo recipients. No other significant differences regarding glucose and lipid parameters between the groups were found [77].

Finally, a 2024 SRMA of 10 RCTs (n = 811), mainly conducted in China, showed that BBR used as an adjunct therapy can significantly improve liver enzyme levels, IR, dyslipidemia, and body weight, with minimal adverse effects. In 7 out of 10 RCTs, patients also presented with comorbid T2D. However, certain results showed increased heterogeneity, highlighting the need for further research. The doses of BBR administered varied between 0.6 and 6.35 g/day, while the study durations ranged from 7 to 24 weeks [81].

*Safety.* BBR has a high safety profile, with fewer side effects than conventional antidiabetic agents [67]. Commonly reported adverse effects include constipation, diarrhea, abdominal pain, and flatulence. In combination therapy with antidiabetic medication, a dose of 300 mg three times daily is well tolerated [68]. BBR carries a low risk of hypoglycemia [67].

*Summary.* BBR is a promising regulator of glucose and lipid parameters in patients with metabolic dysfunction. According to clinical evidence, BBR’s has proven efficient in alleviating glycemic control in patients with T2D is similar to that of metformin and rosiglitazone. In addition, BBR appears to improve several characteristics of MASLD.

### 4.2. Curcumin

Curcumin (CRM) is an NBC derived from the rhizome of *Curcuma longa* (turmeric) that has been extensively studied due to its numerous pharmacological effects. CRM is the main active constituent of turmeric, along with other structurally related curcuminoids. Studies conducted in vitro and in vivo have shown that CRM possesses hypoglycemic, antioxidant, and anti-inflammatory properties, as well as cardio- and hepatoprotective activity. Nevertheless, CRM has low bioavailability, which restrains its clinical use [82]. Therefore, most studies have evaluated different bioavailability-enhanced CRM formulations (e.g., phytosomal, nano-micellar CRM) or CRM in combination with other compounds, e.g., piperine [83] and omega-3 fatty acids [84], which significantly improve CRM absorption or reduce its metabolization [82].

*Clinical evidence in T2D*. According to current evidence, CRM may improve glycemic control and blood lipid profiles in patients with T2D (Table 3). A 2012 RCT conducted by Na et al. involving T2D patients (n = 50) who received 300 mg of curcuminoids daily for 3 months, along with conventional treatment, showed that patients in the interventional group experienced significantly decreased FBG, HbA_1c_, and HOMA-IR levels compared to placebo recipients (n = 50). In addition, in the CRM-treated group, a significant reduction in total free fatty acids and TG levels was observed [85].

An HbA_1c_-lowering effect was also reported by Rahimi et al. (2016) following supplementation with nano-CRM (nano-micelle 80 mg/day for 3 months) versus placebo in 70 patients with T2D. Also, between-group comparisons revealed significant differences in FBG and BMI following supplementation, but no changes were observed in the lipid profile. However, in the nano-CRM-treated patients, significant improvements in the levels of FBG, HbA_1c_, TC, TGs, LDL-c, HDL, as well as in BMI, were observed after the intervention [86].

In another RCT (2017) involving curcuminoids (1000 mg/day, along with piperine 10 mg/day) or a placebo with conventional T2D treatment (n = 100) for 12 weeks, TC, non-HDL-c and lipoprotein (a) levels were significantly reduced in the CRM group at the end of the study. A significant elevation in HDL-c concentration was also reported in CRM-treated patients, although no significant changes in TG and LDL-c levels were observed between the groups [83].

A 2023 updated SRMA involving 28 studies in patients with T2D and MS revealed that CRM supplementation led to significant post-intervention improvements in the levels of FBG, HbA_1c_, LDL-c, HDL-c, and serum insulin. No significant reductions were reported for TC or TG concentrations [93].

Asghari et al. (2024) investigated the effects of nano-CRM, eicosapentaeneoic acid (EPA), and their combination on various metabolic parameters in T2D patients (n = 95). Although significant improvements in insulin, high-sensitivity C-reactive protein (hs-CRP) levels, and TAC were reported after 12 weeks of supplementation with nano-CRM and EPA compared to the placebo, no meaningful differences were observed in FBG, HOMA-IR, quantitative insulin sensitivity check index (QUICKI), and HbA_1c_ levels among the four groups. Nevertheless, HOMA-IR decreased significantly in all treatment groups, while the QUICKI index increased significantly only in the EPA plus nano-CRM group. In addition, HbA_1c_ and TG levels decreased significantly following supplementation with nano-CRM, with or without EPA. The consumption of both EPA and nano-CRM also led to notable improvements in TC and HDL-c levels. In addition, LDL-c decreased non-significantly in all intervention groups [84].

In contrast to these results, Yaikwawong et al. (2024) reported a significant glucose-lowering effect, as evidenced by FPG and HbA1c levels, after CRM supplementation (1500 mg/day) versus placebo for 12 months in T2D patients (n = 227). In addition, CRM-treated patients exhibited significantly lower levels of HOMA-IR and a higher APN concentration [87].

Finally, CRM was proven effective in enhancing vascular health, which, in conjunction with its lipid-modulating potential, decreases cardiovascular risk in T2D patients. According to a 2024 RCT, CRM supplementation (250 mg curcuminoids six times daily for 12 months) exhibited significant reductions in ApoB, LDL-c, small-dense LDL-c, and pulse wave velocity levels, as well as in various proinflammatory cytokines (hs-CRP, IL-6, TNF-α) in T2D patients (n = 227) compared to placebo recipients [88].

*Clinical evidence in MASLD.* CRM represents a promising therapeutic approach for NAFLD patients (Table 3). Rahmani et al. (2016) demonstrated that compared to placebo, CRM intake (500 mg/day of dispersion formulation for 8 weeks) was associated with a significant decrease in HFC in NAFLD patients (n = 77). Also, a significant reduction in FBG, HbA_1c_, TC, TGs, LDL-c, ALT, and AST levels was reported in CRM-treated patients [89].

Improvements in liver fibrosis scores were observed in NAFLD patients supplemented with CRM (500 mg/day, three times daily) in a 2019 RCT. In addition, hepatic steatosis, liver enzymes (ALT, AST), hs-CRP and TNF-α levels, as well as anthropometric indices (BW, BMI, WC), decreased significantly in both groups, but not significantly, suggesting that CRM is not superior to lifestyle modifications in improving inflammation in NAFLD patients [90].

Comparable results were reported by a 2022 RCT involving a similar number of patients with NAFLD and doses of CRM. The findings indicated that, compared to LSI, CRM supplementation did not significantly improve steatosis scores [fatty liver index (FLI) and fatty liver score (FLS)] or adipose tissue-related markers, providing no additional benefits for cardiometabolic health. Nevertheless, the number of patients with severe fatty liver and MS was significantly lower in the CRM group after the intervention compared to the placebo group [91].

CRM supplementation, in combination with piperine (500 mg/day plus 5 mg/day for 3 months) (n = 30), versus a placebo (n = 30) was also not shown to reduce hepatic steatosis and fibrosis in patients with moderate to high NAFLD, but it may be used to improve blood glucose, the lipid profile, anthropometric variables, and liver function [92].

Finally, a 2023 umbrella meta-analysis of patients with NAFLD comprising 11 meta-analyses of 99 RCTs (n = 5546) revealed that CRM was effective in significantly improving levels of ALT, AST, TGs, and HOMA-IR. Also, CRM supplementation has proven effective in reducing obesity [94].

*Safety*. Various animal and human studies have reported the safety and tolerability of CRM, even at high doses (up to 12 g/day via oral administration) [82]. So far, no adverse effects on blood sugar levels have been observed. However, CRM may cause gastrointestinal side effects, such as gastric irritation, flatulence, stimulation of bile flow, and potential cholangitis. In combination with piperine, CRM’s cholecystokinetic effect is amplified, and it may increase the risk of hepatotoxicity [95].

*Summary.* CRM has considerable potential for improving glycemic control, lipid profile, and inflammation status in T2D, with minimal side effects. Although its efficacy in ameliorating MASLD traits is less conclusive, CRM could be recommended as a complementary therapy for these patients. 

### 4.3. Resveratrol

Resveratrol (RSV) is a natural polyphenolic compound initially extracted from the roots of white melon and later identified in many other plants. RSV is considered to possess a broad spectrum of biological activities related to health, exhibiting antidiabetic, anti-obesity, antioxidant, anti-inflammatory, cardioprotective, and antitumor properties, among others [96].

*Clinical evidence in T2D.* Currently, only a small body of RCTs has investigated the effects of RSV supplementation in T2D (Table 4). In a 2012 open-label RCT, it was shown that RSV supplementation (250 mg/day for 3 months), in combination with hypoglycemic medication, led to improvements in HbA_1c_ and TC. However, no significant changes in BW, LDL-c, and HDL-c levels were observed [97].

In another crossover RCT (2016), Thazhath et al. also failed to detect any significant improvements in the glycemic control (FBG, PBG) and BW of diet-controlled T2D patients (n = 14) after 1000 mg RSV/day for two 5-week intervention periods [98].

Timmers et al. (2016) investigated whether RSV intake (150 mg/day) could improve insulin sensitivity in patients with T2D (n = 17). After 30 days of treatment, no changes were reported in hepatic and peripheral insulin sensitivity. The authors argued that the lack of insulin-sensitizing effects following RSV supplementation could be explained by the interaction between RSV and Metformin. Liver fat content also remained unchanged after RSV supplementation, although a negative correlation between plasma RSV levels and intrahepatic lipid content was reported [99].

Similarly, Bo et al. (2016) supplemented T2D patients (n = 179) with two different RSV dosages (500 and 40 mg/day) and a placebo for 6 months but observed no significant improvements in anthropometric indices (body weight, BMI, WC), glucose metabolism parameters (FBG, HbA_1c_, HOMA-IR, insulin), lipid profile (TC, TGs, LDL-c, HDL-c), inflammatory markers (IL-6, APN), or liver enzyme levels (AST, ALT, GGT) between the interventional and control groups. However, a decrease in CRP levels was observed in both RSV-treated arms, although this was not significantly different compared to the placebo. Moreover, a subgroup analysis revealed a decline in CRP levels among patients with a shorter history of T2D following 40 mg/day RSV supplementation. In addition, TC and TG levels were shown to increase modestly in the 500 mg/day RSV-treated patients [100].

By contrast, Hoseini et al. (2019) performed a RCT involving 56 patients with T2D and coronary heart disease, using 500 mg of RSV/day (n = 28) or a placebo for 4 weeks. FBG, IR, and the TC/HDL-c ratio significantly decreased after RSV intake, whereas marked improvements were reported in HDL-c levels, insulin sensitivity, and several biomarkers of oxidative damage. However, RSV supplementation did not alter the IL-1 and TNF-α gene expression [101].

Finally, a significant glucose-lowering effect (FBG, HbA_1c_, HOMA-IR, FI) was reported by Mahjabeen et al. (2022) in T2D patients (n = 110) supplemented with 200 mg RSV/day (n = 55) for 24 weeks compared to a placebo (n = 55). RSV treatment also led to a significantly improved anti-inflammatory status (hs-CRP, TNF-α, IL-6), but no considerable effects were observed on the lipid profile (TC, TGs, HDL-c, and LDL-c) between the two groups [102].

*Clinical evidence in MASLD.* Research on animal models indicates that RSV alleviates fibrosis and inflammation associated with NAFLD [107], but clinical study results are inconclusive (Table 4). Chachay et al. (2014) reported a negative result regarding the antisteatotic effects of RSV. In this study, a large dosage of RSV (3000 mg/day) was administered to patients with NAFLD (n = 10) compared to a placebo (n = 10). No significant improvements were observed in hepatic (AST, ALT), metabolic (FBG, HOMA-IR, insulin, TC, TGs, HDL-c, LDL-c), and antioxidant-related markers following 8 weeks of RSV intake. Moreover, levels of liver enzymes (ALT, AST) increased significantly until week 6 in the RSV-treated group. Nevertheless, RSV was well tolerated. A slight decrease in IL-6 concentration was reported, although repeated measurements at various time points did not reflect significant changes. Other inflammation markers, such as CRP or TNF-α, remained unchanged [103].

In 2015, Faghihzadeh et al. conducted a RCT involving NAFLD patients (n = 50) for 12 weeks using either 500 mg of RSV/day or a placebo, along with lifestyle modifications. ALT levels, as well as hepatic steatosis, decreased significantly in the RSV-treated patients, whereas AST and BMI decreased significantly in both groups, but there was no significant difference between them. Lipid profile and glucose metabolism markers did not differ significantly between the RSV- and placebo-supplemented patients [104].

In contrast to previous results, a consistent reduction in ALT and AST, as well as in glucose, HOMA-IR, TC, and LDL-c levels, was reported after 600 mg RSV/day supplementation (n = 30) for 3 months versus placebo (n = 30) in patients with NAFLD. Inflammation status significantly improved in the RSV-treated patients, as shown by significant changes in TNF-α and APN levels. Cytokeratin 18 M30 (CK18-M30) concentration also declined significantly after RSV intake relative to placebo [105]. However, in a longer-term RCT (2016) involving patients (n = 13) treated with high doses of RSV (1500 mg/day) for 6 months, Heebøll et al. failed to detect any significant effects in attenuating NAFLD-related clinical or histological markers compared to placebo recipients (n = 13) [106].

A lack of antisteatotic effects following RSV supplementation in NAFLD was also reported by a 2021 SRMA, despite significant improvements in inflammatory markers, such as hs-CRP and TNF-α [107].

Finally, a 2021 umbrella review of meta-analyses showed that although RSV supplementation exerts some beneficial effects on glucolipid metabolism in patients with T2D and on inflammation status in patients with NAFLD, the currently existing evidence does not recommend its use in the management of these diseases [108].

*Safety*. Currently, the potential deleterious effects of RSV are not well characterized due to insufficient research. High doses of RSV (2–5 g/day) may cause nausea, hypersensitivity, anal pruritus, and light or mild diarrhea. However, these adverse effects may be insignificant in the healthy state, posing an increased risk particularly for individuals with pathological conditions [109].

*Summary.* The effectiveness of RSV in managing T2D and MASLD has yielded controversial results, making it difficult to reach a conclusion about its therapeutic benefits. However, RSV may alleviate chronic inflammation in patients with metabolic dysfunction. More RCTs with larger samples and longer durations are needed to explain the clinical effects of RSV on glucolipid metabolism parameters.

### 4.4. Anthocyanins

Anthocyanins (ACNs) are a subclass of polyphenols that provide red, purple, and blue colors to fruits and vegetables. ACNs have been reported to exhibit a plethora of therapeutic benefits, including antidiabetic, anti-obesity, anticancer, anti-inflammatory, and antioxidant effects. The latter effects are attributed to the colored pigments found in blackcurrants, berries, and other blue or red fruits [110].

*Clinical evidence in T2D*. ACNs have been found to improve glycemic control and blood lipid profiles in patients with T2D (Table 5) [111]. In 2013, Kianbakht et al. conducted a RCT in T2D patients (n = 37) using ACNs (whortleberry fruit extract, 350 mg every 8 h for 2 months) in combination with antidiabetic medication. At the end of the study, FBG, 2 h PBG, and HbA_1c_ levels significantly decreased in the ACN-treated patients. In addition to ACNs, whortleberry also contains myricetin and chlorogenic acid, which may potentiate its antihyperglycemic effects [112].

Positive effects following pure ACNs supplementation (160 mg twice daily for 24 weeks) were also reported in T2D patients (n = 29) by Li et al. (2015). Compared to placebo (n = 29), ACNs-treated patients experienced lowered FBG, HOMA-IR, TGs, LDL-c, apolipoprotein (apo) B-48, and apo C III levels, as well as increased HDL-c and APN concentrations [113]. Also, purified ACNs supplements administered for 12 weeks were shown to be effective in reducing FBG and serum APN levels relative to placebo in patients with newly diagnosed T2D (n = 62), but not in those with prediabetes (n = 76) [114].

In 2020, Stote et al. conducted an RCT using either 22 g of freeze-dried blueberries or a placebo in 52 patients with T2D. In agreement with previous trials, cardiometabolic parameters, including HbA_1c_, TGs, AST, and ALT, were significantly lower in the experimental group (n = 26), but no significant changes between groups were observed regarding FBG, serum insulin, body weight, LDL-c, HDL-c, and CRP levels [115].

As opposed to the findings mentioned so far, short-term bilberry extract supplementation was not found to be associated with significant improvements in glycemic control, lipid profile, or antioxidant and anti-inflammatory markers in patients with T2D. As such, Chan et al. (2021) reported that the administration of 1.4 g/day of bilberry extract for two 4-week periods did not significantly alleviate cardiometabolic markers (FBG, HbA_1c_, lipid profile, CRP) in ACNs-treated patients compared to placebo recipients [116].

An open-label RCT investigated the effects of a daily intake of 320 mg of ACNs over the course of 4 weeks in T2D patients (n = 12), individuals with prediabetes (n = 14), and healthy individuals (n = 14). Compared to healthy individuals, patients with T2D experienced significant improvements in IL-6 and TNF-α levels. However, the trial did not employ a placebo, and the T2D participants followed an anti-inflammatory diet, which might have enhanced the effects of ACNs [117].

A 2023 SRMA by Mao et al. involving patients with T2D analyzed 13 RCTs (n = 703) and concluded that an average intake of 320 mg of ACNs, either from fruit extracts or pure supplements, over approximately 8 weeks led to significant reductions in FBG, 2 h PBG, HbA_1c_, TG, and LDL-c levels. Nevertheless, no significant effects were observed regarding HOMA-IR, FI, TC, HDL-c, or blood pressure levels in patients with T2D. Notably, compared to pure supplements, ACNs derived from fruit extracts or powders exhibited a more pronounced effect on HbA_1c_ levels [111].

*Clinical evidence in MASLD.* Several RCTs (Table 5) demonstrated a significant reduction in liver enzyme levels following ACNs intake in patients with NAFLD [119]; however, other human studies found no beneficial effects of ACNs on these parameters [120]. Chang et al. reported in 2014 that *Hibiscus sabdariffa* extract consumption for 12 weeks had some beneficial effects on liver steatosis, mainly through improvements in anthropometric indices and free fatty acid levels. The latter decreased significantly in the intervention group compared to the placebo at the end of the follow-up period. The fatty liver score improved in both groups but did not differ significantly between HSE- and placebo-treated patients after 12 weeks. Also, no significant changes between groups were observed regarding ALT and AST levels, glucose concentration, or lipid profile [118].

In 2015, Zhang et al. showed that compared to a placebo, purified ACN intake (320 mg/day for 12 weeks) led to a significant reduction in plasma ALT concentration and 2 h glucose loading test levels in patients with NAFLD. Improvements in FBG, HOMA-IR, TGs, and HDL-c were also shown, but mean percentage changes were not significantly different from controls. ACN supplementation did not affect AST, FBG, plasma insulin, TC, and LDL-c levels or anthropometric indices. However, the ACN-treated patients exhibited significant decreases in cytokeratin-18 M30 fragment (CK18-M30) levels, which is a predictor of NAFLD progression, and myeloperoxidase concentration, which is an inflammation- and oxidative stress-related enzyme, as well as a tendency toward improvement in the NAFLD fibrosis score [119].

Sangsefidi et al. (2021) tested the effects of *Cornus mas* L. fruit extract (20 mL daily for 12 weeks) on several NAFLD-related markers. The authors failed to detect any significant differences within or between the intervention group (n = 22) and the placebo controls (n = 18) regarding ALT and AST levels or steatosis scores. Despite a significant decrease in CK18-M30 concentration in the ACN-supplemented NAFLD patients, no significant difference between groups was found at the end of the study period [120].

Another RCT (2024) involving 87 patients with metabolic dysfunction-associated fatty liver disease (MAFLD) reported that lyophilized *Cornus mas* L. fruit powder (CMFP), with/without diet therapy, led to statistically significant decreases in liver enzyme levels glycemic indices, lipid profile and anthropometric variables from baseline to the end of the study period. CRP levels also decreased significantly at the end of the follow-up period (8 weeks) in the groups treated with CMFP and diet therapy, and only diet therapy. Notably, no significant differences in anthropometric, biochemical, and inflammatory parameters were reported at the end of the study between the CMFP with diet therapy group and the diet therapy alone group [121].

Finally, Khan et al. concluded in their 2024 SRMA that there is currently no evidence that ACN supplementation could significantly improve liver function in patients with NAFLD [122]. These results are in line with another 2024 SRMA, which found no significant effects on liver function following supplementation with *Cornus mas* L. in individuals at high risk [123].

*Safety*. So far, no adverse effects associated with ACNs consumption have been reported. In various human studies, most subjects tolerated a dosage of 160 mg of ACNs extract administered twice daily for 2 months. Only 4% of participants experienced side effects, which consisted of eczema and gastrointestinal distress [124].

*Summary*. RCTs investigating the effects of ACNs supplementation on cardiometabolic risk factors in patients with T2D or MASLD are scarce. While ACNs may play a role in the management of T2D via improvements in glycemic indices, lipid profiles, or chronic inflammation, there is a lack of evidence regarding the extension of these effects to patients with MASLD.

### 4.5. Catechins

Catechins are a type of natural phenolic compound found in high concentrations, particularly in green tea, but also in black and oolong tea. The main catechins in green tea extracts include epigallocatechin gallate (EGCG), epigallocatechin, epicatechin gallate, and epicatechin. Among these, EGCG is the most abundant catechin (50–80%) [125] and is often studied due to its antioxidant, anti-inflammatory, antimutagenic [126], and cardioprotective properties [125].

*Clinical evidence in T2D.* Research outcomes regarding catechins’ ability to improve glycemic control and blood lipid levels are heterogeneous (Table 6). In 2009, green tea enriched with either 582.8 mg of catechins (experimental group, n = 23) or 96.3 mg of catechins/day (control group, n = 20) was administered to patients with T2D. After 12 weeks, a significant decrease in HbA_1c_ levels was observed in the catechin group patients receiving insulinotropic agents compared to controls. Also, in these patients (approximately 77%), insulin levels significantly increased at the end of the study period, suggesting catechins’ capacity to stimulate insulin secretion. The catechin-rich beverage intake led to a decrease in abdominal obesity, as evidenced by a significantly lower WC in patients assigned to the intervention group after 12 weeks. Similarly, APN levels increased significantly in the intervention group from baseline to the end of the study, although there was no significant difference between groups [127].

Human RCTs have not always demonstrated the benefits of catechin supplementation in the management of glycemic control and lipid profiles. A double-blind, placebo-controlled trial (2011) reported that in T2D patients with obesity, a daily dose of decaffeinated green tea (GTE) extract (856 mg EGCG) for 16 weeks led to no statistically significant improvement between groups in markers of glucolipid metabolism. However, a significant within-group reduction of 0.4% in HbA_1c_ level was observed in supplemented patients at the end of the follow-up period. GTE-treated patients also experienced a significant decrease in HOMA-IR and WC [128].

In a 2013 2-month trial, patients with T2D (n = 63) were randomly assigned to three groups with daily intakes of green tea as follows: four cups (n = 24), two cups (n = 25), and a control group (n = 14). Except for BW, BMI and WC, which significantly decreased in the four cups of green tea group, no significant changes in glucolipid or oxidative stress parameters were detected in the other two groups or between groups [129].

In 2014, Liu et al. performed an RCT involving 92 patients with T2D and lipid disturbances, who received either 500 mg of GTE three times daily (n = 39) or a placebo (n = 38) for 16 weeks. GTE consumption significantly decreased TG and HOMA-IR levels while increasing HDL-c concentration at the end of the intervention. APN increased pronouncedly in both groups, without showing a statistically significant difference between them. In addition, between-group comparisons only revealed a decreasing tendency for TG levels, with no statistically significant differences after 16 weeks of GTE versus the placebo [125].

In a double-blind, placebo-controlled RCT (2020), 44 patients with T2D were supplemented with either two tablets of EGCG (n = 25) or a placebo for 2 months. A significant reduction in the mean levels of TC and TGs was observed in the EGCG-treated patients. Also, compared to the placebo group, the mean changes in TC and total antioxidant capacity were significantly different in the intervention group. No other statistically significant differences were found in the lipid profile between the two groups. IL-6 levels remained unchanged [130].

In 2023, an RCT was performed involving patients with T2D and nephropathy to investigate the effects of green tea infusion on metabolic parameters. Patients were randomly divided into three groups: one receiving three cups of green tea/day (n = 22), another receiving two cups of green tea/day (n = 22), and a control group (n = 20). Results showed that, compared to the control group, patients consuming three cups of green tea daily presented significant improvements in HbA_1c_, TC, and HDL-c levels after 12 weeks [131].

Finally, an SRMA performed by Asbaghi et al. (2020) involving 14 RCTs concluded that green tea supplementation exerted no effects on FBG, FI, HbA_1c_, and HOMA-IR in patients with T2D. Study participants were supplemented with green tea, GTE, or EGCG in doses ranging from 300 to 10,000 mg/daily for 8–16 weeks. However, subgroup analysis indicated that green tea intake for more than 8 weeks significantly decreased FBG, while supplementation for 8 weeks or less led to a significant decrease in HbA_1c_. An improved glycemic response after green tea intake was reported exclusively by RCTs performed in Asia, while those conducted on other populations did not show significant outcomes. Inter-ethnic differences may be explained by a genetic polymorphism responsible for a slower metabolism of green tea among Asian individuals, which enhances its glucose-lowering effect [132].

*Clinical evidence in MASLD.* In a 2013 randomized double-blind study, green tea rich in catechins (700 mL/day containing > 1 g catechins), green tea low in catechins, or a placebo were administered to patients with NAFLD (n = 17). After 12 weeks, ALT levels and body fat percentage (BF%) significantly decreased in the green tea rich in catechins group compared to the other two groups. Also, HFC was significantly improved in the green tea rich in catechins group compared with the low-density catechin and placebo groups, as indicated by the liver-to-spleen attenuation ratio [133].

**Table 6:** Summary of RCTs investigating the hypoglycemic, hypolipidemic, and anti-steatosis effects of catechins.

Disease	Participants (Total)	Intervention	Control	Duration	Main Outcomes */**	References
T2D	n = 43	Catechins 582.8 mg/day	Catechins 96.3 mg/day	12 weeks	↓ HbA_1c_, WC, ↑ insulin *	[127]
T2D	n = 68	GTE 1500 mg/day	Placebo	16 weeks	↓ HbA_1c,_ HOMA-IR, WC **	[128]
T2D	n = 63	Green tea 2 or 4 cups/day	No green tea	2 months	↓ BMI, BW, WC **	[129]
T2D	n = 92	GTE 1500 mg/day	Placebo	16 weeks	↓ HOMA-IR, TGs, ↑ HDL-c, APN **	[125]
T2D	n = 44	EGCG 300 mg/day	Placebo	2 months	↓ TC, ↑ TAC *	[130]
T2D	n = 64	Green tea 3 or 2 cups/day	No green tea	12 weeks	↓ HbA_1c_, TC, ↑ HDL-c *	[131]
NAFLD	n = 17	High-density catechins- green tea	Placebo	12 weeks	↓ BF, ALT, HFC *	[133]
NAFLD	n = 71	GTE 500 mg/day	Placebo	90 days	↓ BW, BMI, ALP *	[134]
NAFLD	n = 45	GTE 550 mg/day + diet	Placebo	3 months	↓ BW, AST, FBG *	[135]
NAFLD	n = 80	GTE 1000 mg/day	Placebo	12 weeks	↓ BW, BMI, ALT, AST, TC, TGs, LDL-c, HDL-c, HOMA-IR, hs-CRP, APN *	[136]

* Statistically significant between groups (*p* < 0.05); ** Statistically significant from baseline (*p* < 0.05); “↓”, decreased; “↑”, increased. Abbreviations: ALT, alanine aminotransferase; ALP, alkaline phosphatase; APN, adiponectin; AST, aspartate aminotransferase; BMI, body mass index; BF, body fat; BW, body weight; FBG, fasting blood glucose; GTE, green tea extract; HbA_1c,_ glycosylated hemoglobin; HDL-c, high-density lipoprotein cholesterol; HFC, hepatic fat content; HOMA-IR, homeostatic model assessment of insulin resistance; hs-CRP, high-sensitivity C-reactive protein; LDL-c, low-density lipoprotein cholesterol; TAC, total antioxidant capacity; TC, total cholesterol; TGs, triglycerides; WC, waist circumference.

Later, in 2016, Pezeshki et al. demonstrated that GTE (500 mg/day for 12 weeks) led to significant improvements in ALT, AST, and ALP levels in patients with NAFLD (n = 35). In the placebo group (n = 36), ALT and AST also declined, but to a lesser extent, while ALP showed a significant reduction at the end of the study. Notably, BW decreased significantly in both groups after 90 days, but the mean weight change was significantly greater in the GTE group compared to the placebo group [134].

Similarly, following 550 mg GTE intake and diet therapy for 3 months, patients with NAFLD (n = 21) experienced significant improvements in BMI, AST, and FBG levels, as well as in BW, compared to placebo recipients (n = 24). However, no significant differences between groups were observed regarding ALT and HOMA-IR levels [135].

Compared to placebo (n = 40), GTE administration (1000 mg/day for 12 weeks) to patients with NAFLD and DLD (n = 40) was found to be effective in improving BW, BMI, ALT, AST, TC, TGs, LDL-c, HDL-c, and HOMA-IR. Also, GTE significantly improved inflammatory markers, specifically hs-CRP and APN, and fatty liver grading [136].

A 2020 SRMA including 15 RCTs reported that although the overall effect of green tea on liver enzymes was not significant, the subgroup analysis revealed that it may decrease liver enzyme levels in NAFLD patients. However, in healthy subjects, a modest but significant increase in hepatic enzyme concentration was observed [137].

*Safety*. Green tea intake raises liver-related safety concerns due to the potential risk of hepatotoxicity [138]. According to the European Food Safety Authority (EFSA), the intake of doses equal to or above 800 mg EGCG/day as a food supplement may cause a significant increase in serum transaminase levels [139]. A 2016 SRMA concluded that liver-related adverse effects after green tea extract intake are expected to be rare [138].

*Summary*. Green tea exerts important anti-inflammatory and antioxidant effects that may benefit patients with T2D and MASLD. Nevertheless, the evidence regarding the effectiveness of green tea in improving glycemic control and liver steatosis is not conclusive.

### 4.6. Other Phenolic Compounds and Carotenoids

Intake of dietary phenols and carotenoids derived from artichokes, tomatoes, and citrus fruits has been shown to exert various biological effects, particularly hypoglycemic [140,141,142], hypolipidemic [141], and hepatoprotective [143] effects.

Artichoke (*Cynara cardunculus* var. *scolymus* L.) has long been used as an herbal remedy with powerful therapeutic properties. Artichoke leaf extract (ALE) mainly consists of phenolic acids, sesquiterpene lactones, and flavonoids [140]. Lycopene (LYC), a lipophilic carotenoid, is found naturally in red-colored fruits such as tomatoes, red grapefruit, and watermelon. LYC’s antioxidant capacity is twice as effective as that of ß-carotene and 100 times more efficient than that of α-tocopherol [142]. Citrus flavonoids (hesperidin) are abundant in phenolic compounds with enhanced antioxidant activity that can improve glucolipid parameters [144].

*Clinical evidence in T2D.* There are limited published data linking artichoke or ALE to glycemic control (Table 7). In 2012, Fallah Huseini et al. performed an RCT involving 72 patients with T2D who were supplemented with either free-fiber ALE (1200 mg/day) (n = 36) or a placebo (n = 36) for 2 months, along with their standard anti-T2D medication. ALE was found to be efficient in alleviating certain lipid profile markers (TC, LDL-c) compared to placebo, but did not lead to significant changes in glycemic parameters (FBG, 2 h PBG, HbA_1c_), liver enzymes (ALT, AST), or other lipids. The authors suggested that dietary fibers may be responsible for improving glycemic control in patients with T2D [145].

More recently, Ebrahimi-Mameghani et al. also failed to detect significant differences in glucolipid parameters (FBG, HOMA-IR, TC, LDL-c, HDL-c), except for TGs, following ALE supplementation (1800 mg/day for 12 weeks) compared to a placebo in patients with MS. However, ALE led to a significant decline in HOMA-IR and insulin levels in a subgroup of patients with MS and TCF7L2 polymorphism (n = 10). The latter is associated with an increased risk of developing T2D and could explain the variation in metabolic benefits of ALE among different subjects [140].

In a 2000 RCT, 52 patients with well-controlled T2D who supplemented with placebo for 4 weeks were randomly assigned to receive tomato juice (n = 15), vitamin E (n = 12), vitamin C (n = 12), or placebo (n = 15) for another 4 weeks. Results showed that levels of LYC increased significantly (almost three-fold) following supplementation with tomato juice. Also, these patients exhibited an increased resistance to LDL oxidation (~42%), similar to that observed in the vitamin E-treated patients (54%). However, plasma CRP levels remained unchanged after tomato juice supplementation, while they declined significantly among patients receiving vitamin E. No significant changes were reported regarding BMI, FBG, and TC levels after tomato juice consumption [146].

However, Homayouni et al. (2018) demonstrated that 500 mg/day HES supplementation is effective in improving the inflammation status of patients with T2D. As such, oral HES intake led to significant changes in the mean percent changes of TNF-α, IL-6, and hs-CRP levels in patients with T2D (n = 32) compared to placebo recipients (n = 32) after 6 weeks [147].

In a 2020 RCT, 49 patients with MS were randomly assigned to receive either HES (1000 mg/day) or a placebo, along with lifestyle modifications. After 12 weeks, changes in lifestyle led to a significant reduction in BW in both groups. In addition, FBG, TG, and TNF-α levels decreased significantly in the HES group (n = 24) compared to the placebo group (n = 25). Compared to baseline, hs-CRP, glucose, insulin, TC, LDL, and TG concentrations declined significantly after HES supplementation [148].

Finally, a group-specific meta-analysis involving 152 patients with T2D (2022) showed that LYC supplementation is effective in improving FBG [142]. However, a 2019 SRMA that included six RCTs (n = 318) investigating the effect of HES supplementation on glycemic control failed to detect any significant improvement in glycemic indices [FBG, HbA_1c_, HOMA-IR, quantitative insulin sensitivity check index (QUICKI), or insulin] in healthy overweight individuals or subjects with either prediabetes or T2D [153].

*Clinical evidence in MASLD.* Phenolic compounds have also demonstrated therapeutic potential in individuals with MASLD/MASH (Table 7). In 2016, 60 patients with NASH were enrolled in an RCT to receive either *Cynara scolymus* extract (2700 mg/day) or a placebo for 2 months. Compared to the placebo, significant improvements in ALT, AST, TC, and TG levels were reported at the end of the study [149].

Panahi et al. (2018) showed that, compared to placebo (n = 41), supplementation with ALE in patients with NAFLD (n = 49) led to a significant reduction in serum ALT and AST, as well as improvements in the ALT/AST ratio, portal vein diameter, hepatic vein flow, and liver size. Also, between-group comparisons revealed that ALE-treated individuals experienced significantly lower levels of TC, LDL-c, TGs, and HDL-c. Notably, ALE supplementation had no significant effect on glycemic control (FBG, HbA_1c_, insulin) [143].

*Cynara cardunculus* (Cyc) has also been found to exert anti-steatosis effects, particularly in combination with bergamot phenolic fraction (BPF). The beneficial influence of bergamot polyphenol extract against NAFLD was demonstrated in a previous in vitro study [154]. In a 2020 RCT, 80 patients with T2D and NAFLD were randomly allocated into four groups to receive Cyc—300 mg/day (n = 20); BPF—300 mg/day (n = 20); Cyc plus BPF (50/50%)—300 mg/day (n = 20); or a placebo (n = 20). Supplementation with both Cyc and BPF resulted in improvements in liver imaging profiles and several markers of liver fibrosis in NAFLD associated with T2D. Also, compared to placebo, Cyc + BPF significantly decreased serum concentrations of ALT, AST, GGT, ALP, and TNF-α, as well as markedly improved several markers of oxidative stress, such as GSH-Px, SOD, and MDA [150].

In addition, a 2021 SRMA that included eight RCTs concluded that artichoke supplementation significantly reduced liver enzyme levels compared to placebo, particularly in individuals with NAFLD. The doses of artichoke ranged from 100 to 2700 mg/day, whereas the study duration varied from 4 to 12 weeks [155].

The hepatoprotective effect of HES was investigated in a 2019 RCT involving 49 NAFLD patients who received either HES (1 g/day) (n = 24) or a placebo (n = 25) for 12 weeks. All participants were instructed to follow lifestyle changes during the study duration. At the end of the study, reductions in ALT, GGT, TC, TGs, hs-CRP, TNF-α, and the steatosis score were significantly improved in the HES-treated group compared to the placebo group. FBG decreased significantly in the interventional group, but this change was not significantly different from that in the placebo group. HES supplementation alongside lifestyle adjustments has proven superior to lifestyle changes alone in alleviating NAFLD [151].

In line with these results, Yari et al. demonstrated in a 2020 open-label RCT that HES in combination with flaxseeds ameliorates hepatic steatosis, glucolipid metabolism, and inflammation in patients with NAFLD. Study participants were divided into four groups, with three groups receiving either HES (1 g daily) (n = 22), flaxseeds (30 g daily) (n = 24), or HES in combination with flaxseeds (1 g HES and 30 g flaxseeds daily) (n = 25), and one group receiving no supplementation (n = 21). All participants were given lifestyle recommendations. Liver steatosis declined significantly in all groups at the end of the study, with no difference in the mean reduction of the controlled attenuation parameter score (CAP). Liver fibrosis decreased significantly among flaxseed-supplemented patients, with or without HES. Lifestyle modifications and dietary supplements were more effective in reducing HFC than LSI alone, as evidenced by significant differences in the fatty liver score. ALT and GGT levels reduced significantly in all groups, but not in the control group. The lipid profile (TC, TGs, and LDL-c) improved significantly in all intervention groups compared to baseline, while glycemic control (FBG, HOMA-IR, insulin) was significantly ameliorated in the flaxseed groups, with or without HES, compared to the control group. Finally, hs-CRP and TNF-α decreased significantly within the HES groups, alone or in combination with flaxseeds, after 12 weeks [152].

*Safety.* When used in recommended amounts or as a standardized extract, ALE is well tolerated with minimal side effects. Moderate transient mild effects, such as increased flatulence, were reported by 1 out of 100 participants in a study investigating its safety [156]. LYC, consumed either through supplements or diet, has also been shown to be safe and well tolerated at different concentrations [157]. Finally, HES is considered a generally safe NBC, even when administered at very high doses, but more research is needed to evaluate its safety in clinical settings [158].

*Summary.* Currently, there is limited clinical evidence supporting the therapeutic role of ALE, LYC, and HES in patients with T2D. More large-scale, well-designed RCTs are needed to validate their clinical effectiveness, particularly for LYC, where a significant shortage of RCTs exists. The addition of dietary fibers to phenolic compounds may enhance their hypoglycemic effects observed in both T2D and MASLD. ALE may also help mitigate liver steatosis, inflammation, and oxidative stress.

### 4.7. Silymarin

Milk thistle fruits contain a blend of four major flavonolignans collectively referred to as silymarin (SLM) [159], with silybin (or silibinin) as the primary component [160]. SLM has been shown to possess a wide range of biological activities, encompassing antioxidant, anti-inflammatory, lipid-lowering, antitumor, immunomodulating [159], anti-fibrotic, antidiabetic, choleretic, and liver-regenerating properties [160].

*Clinical evidence in T2D*. Currently, only a few RCTs are available involving individuals with T2D, most of which show that SLM, in combination with standard treatment or aerobic training, improved glucolipid metabolism parameters (Table 8). Huseini et al. (2006) conducted an RCT with 51 patients aged between 40 and 65 years who had a history of T2D for more than 2 years. Patients assigned to the experimental group (n = 25) received 200 mg SLM three times daily, in addition to standard treatment, for 4 months. The results showed that SLM intake, compared to both baseline and placebo, led to significant reductions in FBG, HbA_1c_, TC, TGs, LDL-c, ALT, and AST levels [161].

Ebrahimpour-Koujan et al. investigated the effects of SLM extract supplements in 40 subjects with T2D in 2015 and again in 2018. In both triple-blinded RCTs, patients with T2D received either 140 mg of SLM thrice daily (n = 20) or a placebo (n = 20) for 45 days. The results of the first study showed that SLM supplementation led to a significant decrease in anti-inflammatory and oxidative stress markers. Specifically, hs-CRP significantly decreased, while GPX and SOD activity, as well as TAC, significantly increased in the SLM-supplemented patients compared to the placebo group. MDA declined significantly compared to baseline following SLM consumption [162].

The second RCT revealed that compared to placebo, SLM significantly improved FBG, serum insulin, HOMA-IR, TGs, HDL-c, and QUICKI levels. Also, in the experimental group, a significant decrease from baseline was observed in TC and LDL-c levels [163].

In 2020, Ghalandari et al. performed a RCT involving 60 men with T2D, who were randomly assigned to four groups: one receiving 140 mg/kg SLM daily, one receiving 140 mg/kg SLM daily plus aerobic training (AT), one receiving AT plus a placebo, and one receiving a placebo. The AT protocol included three training sessions per week, with a duration of 20–45 min at an intensity of 60–80% of the heart rate reserve. Results showed that FBG, insulin, and HOMA-IR levels were significantly reduced at the end of the study in all intervention groups, except the control group. SLM and AT were significantly more effective in reducing FBG levels than SLM alone. AST, ALT, and ALP levels declined significantly in all three experimental groups compared to baseline and the control group. The reduction of AST was significantly lower in the SLM plus AT group compared to the SLM group. BW and weight-to-hip ratio (WHR) decreased significantly compared to baseline in the SLM plus AT and AT groups. [164].

In contrast to these results, a 2024 open-label RCT conducted by Ferdowsi et al. involving 48 patients with T2D indicated that the intake of 140 mg SLM (three capsules daily) plus diet for 12 weeks failed to significantly decrease the levels of FBG, HbA_1c_, and blood lipid markers. The comparison intervention was diet [165].

*Clinical evidence in MASLD.* SLM has traditionally been used for its potential to alleviate liver diseases. However, RCTs investigating its efficacy in NAFLD management are scarce and have yielded inconclusive results, especially when SLM is used as monotherapy [169] (Table 8). Aller et al. (2015) investigated the effects of SLM in combination with vitamin E (n = 36) and lifestyle modification on glucolipid metabolism markers, liver enzyme levels, several noninvasive NAFLD parameters, and anthropometric indices before and after the intervention. The results showed that, after the intervention, glucose, HOMA-IR, and ALT levels declined significantly compared to baseline only in the control group. However, a significant decrease in GGT level was observed in both groups. No changes were reported in the levels of TGs. Both groups experienced significantly improved liver function tests (FLI and noninvasive NAFLD index) and anthropometric measurements (BW, WC). Notably, patients in the intervention group who failed to lose 5% of BW still exhibited decreased GGT levels and improvements in liver function, while patients in the control group who did not lose 5% of their weight displayed no changes in metabolic status [166].

In agreement with these results, in another RCT (2019), 150 patients with NAFLD were assigned to five groups: SLM 140 mg/day (n = 30), metformin 500 mg/day (n = 30), pioglitazone 15 mg/day (n = 30), vitamin E 400 IU/day (n = 30), and lifestyle plus a placebo (n = 30). All patients were given lifestyle advice during the study duration. ALT, AST, TGs, and LDL-c declined significantly in all treatment groups. Also, significant improvements in FBG, TC, BMI, and WC were reported in all groups after 3 months. Between-group comparisons revealed that ALT and AST improved significantly in the pioglitazone, metformin, and SLM groups compared to the other two groups, while TC decreased significantly in the placebo and vitamin E groups compared to the other three groups [167].

However, in a 2017 RCT involving 99 patients with NASH, which is a more severe form of NAFLD, an SLM intake of 700 mg three times daily for 48 weeks (n = 49) did not achieve a significant reduction in the NAS score (the sum of scores for steatosis, lobular inflammation, and ballooning) compared to the placebo (n = 50). However, there was a significant reduction in liver fibrosis in the SLM-treated group compared to the placebo. Also, a significantly greater percentage of individuals in the interventional group experienced improvements in liver fibrosis and stiffness compared to placebo recipients. Within-group comparisons showed that SLM-treated patients displayed a significant decline in ALT, AST, GGT, HbA_1c,_ and TGs. Except for TGs, other parameters did not change significantly in the interventional group compared to placebo. HDL-c concentration also increased significantly in the SLM group, whereas a significant reduction in ALT levels was reported in the placebo controls [168].

In a 2024 RCT, Jin et al. demonstrated that, compared to placebo (n = 41), SLM supplementation (103.2 mg/day) significantly decreased liver fibrosis (assessed by liver stiffness measurement), GGT, and ApoB levels, but it had no significant effect on liver steatosis (as measured by CAP), fibrosis index, aminotransferases, glucolipid parameters, anthropometric indices, hs-CRP, and SOD levels in patients with MASLD (n = 42) [169].

Finally, a meta-analysis involving six RCTs concluded that although SLM supplementation significantly reduced the levels of AST and ALT in NAFLD patients, the results were clinically irrelevant. No significant change in GGT levels was observed following SLM supplementation [170].

*Safety*. SLM utilization has proven to be generally safe and well tolerated, with minimal adverse effects in both T2D [171] and MASLD/MASH [170].

*Summary*. The effect of SLM supplementation in patients with T2D remains inconclusive, although it appears to improve glycemic parameters. SLM seems effective in decreasing liver enzyme levels in MASLD, but these findings warrant cautious interpretation, particularly in clinical practice.

### 4.8. Cinnamon

Cinnamon (CNM) has long been a popular spice, with four of the 250 species belonging to the genus Cinnamomum used for this purpose. CNM is available in many forms, such as sticks (bark), pulverized stick powder, and extracts derived from the powder, each with a different phytochemical composition and bioavailability. Currently, it is not clear which bioactive compounds are responsible for CNM’s biological effects, making standardized formulation difficult to develop [172]. It has been shown that CNM has hypoglycemic, hypolipidemic, anti-inflammatory, antioxidant, and antihypertensive properties [173].

*Clinical evidence in T2D.* Oral supplementation of CNM supplements has been suggested to be effective in improving glycemic control and lipid profile, possibly providing a potential adjuvant for the treatment of T2D. However, RCT results are conflicting, particularly regarding CNM’s lipid-lowering properties (Table 9). In a 2003 placebo-controlled RCT, Khan et al. reported that following supplementation with 1, 3, or 6 g of CNM/day for 40 days, patients with T2D receiving sulfonylureas (n = 30) experienced a significant reduction in serum glucose, TGs, and TC levels. A significant change was also observed in LDL-c concentration in patients consuming 3 and 6 g of CNM [174].

Akilen et al. (2010) demonstrated that, compared to placebo (n = 28), 2 g of CNM for 12 weeks led to a significant decrease in mean HbA_1c_ in patients with poorly controlled T2D (n = 30). In addition, a significant reduction in FBG, BMI, and WC was observed in the interventional group compared to baseline, but CNM supplementation had no effects on blood lipid parameters (TC, TGs, HDL-c, LDL-c), either within or between the groups [175].

Lu et al. randomly assigned 66 patients with T2D into three groups: one receiving 120 mg of CNM/day (n = 23), another receiving 360 mg of CNM/day (n = 23), and a placebo group (n = 20). All patients were treated with gliclazide throughout the trial. After 3 months, FBG and HbA_1c_ were significantly reduced in the CNM-treated patients. TG levels also decreased significantly in the group receiving 120 mg of CNM/day. TC, LDL-c, HDL-c, and liver enzyme levels (ALT, AST) remained unchanged in all patients at the end of the follow-up period [176].

Vafa et al. (2010) tested the effects of CNM supplementation (3 g/day for 8 weeks) compared to a placebo in 37 patients with T2D. Compared to baseline, levels of FBG, HbA_1c_, and TGs, as well as anthropometric indices (BMI, BW, BF%), significantly improved in the CNM-supplemented group. However, no significant differences were observed between the groups regarding glucolipid or anthropometric parameters [177].

In 2019, Zare et al. conducted a triple-blind, placebo-controlled RCT involving 138 patients with T2D, divided into four groups based on their BMI (>/< 27 kg/m^2^) and intervention (1 g of CNM/day or placebo for 3 months). The results showed that CNM improved glycemic (FBG, 2 h PBG, HOMA-IR, FI) and lipid (TC, LDL-c, HDL-c) measurements, except for TG levels. In addition, anthropometric indices (BMI, BF%, visceral fat) also significantly ameliorated in the CNM-supplemented patients. Improvements were significantly greater in patients with BMI > 27 kg/m^2^, except for TC and LDL-c [178].

Another RCT (2020) involving 39 patients with T2D (intervention, n = 20 and placebo, n = 19) investigated the effect of CNM supplementation on inflammation status. No beneficial impact was observed on plasma levels of hs-CRP, IL-6, or TNF-α following CNM consumption. Moreover, a significant within-group reduction in hs-CRP was reported in the placebo controls at the end of the study [179].

Finally, a review analyzing 11 RCTs (n = 694) concluded that CNM supplementation, in addition to hypoglycemic medication and lifestyle changes, has only modest effects on glycemic control (FBG and HbA_1c_) in patients with T2D [172]. Another clinical review reported that supplementation with 1 to 6 g of CNM seemed to be beneficial in alleviating glucose metabolism parameters [181]. In addition, a 2025 SRMA including 28 RCTs (n = 3054) reported, with significant heterogeneity, that CNM appears to improve BMI, glycemic, and lipid parameters in patients with T2D, especially when administered in capsule form at a daily dosage of ≤2 g [182].

*Clinical evidence in MASLD.* CNM’s hepatoprotective effects in patients with NAFLD were investigated in a single double-blind, placebo-controlled RCT (2014) (n = 45). All patients followed lifestyle recommendations throughout the study. The authors reported that CNM supplementation (1.5 g/day for 12 weeks) led to significant improvements in ALT, AST, GGT, TC, and TG levels compared to placebo. No changes were reported regarding HDL-c concentration. In addition, significant reductions in FBG, HOMA-IR, QUICKI, and hs-CRP concentrations were observed in the intervention group compared to the control group. LDL-c decreased significantly in both groups. BMI and WC remained unchanged during the study [180].

*Safety*. CNM appears to be well tolerated, without adverse effects, and may improve health status as an adjunct treatment for T2D. However, further high-quality studies are needed to firmly establish its risk potential and safety profile [183].

*Summary*. CNM may be employed as an add-on phytotherapy to standard medication in T2D, particularly due to its glucose-lowering effect. However, RCTs have not always shown positive outcomes, and it seems that CNM’s therapeutic potential in glycemia regulation is rather modest. Regarding MASLD, there is currently insufficient clinical evidence to recommend the use of CNM in its management.

### 4.9. Fenugreek (Trigonella Foenum-Graecum)

Trigonella foenum-graecum (TFG), or fenugreek, has been widely used for its antidiabetic properties, primarily derived from its seeds. The hypoglycemic effect of TFG is attributed to several NBCs, including trigonelline, diosgenin, soluble fibers, and 4-hydroxyisoleucine. In addition, TFG has demonstrated anti-hyperlipidemic, anti-obesity, anti-inflammatory, antioxidant, and anticancer effects [184].

*Clinical evidence in T2D*. In recent years, multiple RCTs have investigated the role of TFG in T2D management (Table 10). In 2001, Gupta et al. performed a double-blind placebo-controlled RCT in newly diagnosed patients with T2D (n = 25), testing TFG supplementation (1 g/day of hydroalcoholic extract for 2 months, n = 12) on glycemic parameters. Although the results failed to show a significant difference between groups regarding FBG and 2 h PBG levels, TFG supplementation has proven effective in improving insulin resistance, as well as TG and HDL-c levels [185].

In 2008, Lu et al. evaluated the effectiveness of TFG extract combined with sulfonylureas (SU) for managing T2D in 69 patients with insufficiently controlled T2D using SU alone. Patients received either TFG (n = 46) or placebo (n = 23) daily for 12 weeks in conjunction with SU. All participants were instructed to maintain lifestyle recommendations throughout the study. At the end of the follow-up period, TFG-treated patients experienced a significant reduction in FBG, 2 h PBG, and HbA_1c_ compared to both baseline and placebo. No improvements in BMI levels were observed in either group [186].

Similar results were reported by Rafraf et al. (2014) in a triple-blind RCT involving 88 patients with T2D. After 8 weeks of supplementation with TFG (10 g/day of powdered seeds), FBG, HbA_1c_, serum insulin, HOMA-IR, TC, TG, and APN levels significantly improved in the interventional group compared to the placebo. No changes were observed in LDL and HDL-c levels [187].

In a 12-week open-label trial involving nine patients with T2D who were not adequately controlled with metformin, participants consumed either TFG (2 g/day) or glibenclamide (5 mg/day). It was shown that TFG led to a significant increase in FI concentration and the HDL/LDL ratio, but did not significantly affect FBG, HbA_1c_, HOMA-IR, TG, or LDL-c levels. However, compared to TFG, glibenclamide significantly lowered HOMA-IR and HbA_1c_ levels. Notably, HbA_1c_ levels were lower at baseline in the glibenclamide-treated group compared to the TFG-supplemented group [188].

Geberemeskel et al. (2019) investigated the effect of TFG supplementation (25 g of seed powder solution twice daily) versus metformin on the lipid profile in patients with newly diagnosed T2D (n = 95). After 1 month, in TFG-treated patients (n = 49), significant improvements in TC, TG, and HDL and LDL cholesterol levels were observed compared to both baseline and the control group. Blood lipid levels in the control group remained unchanged [189].

Hadi et al. (2020) reported that, in patients receiving standard T2D medication, supplementation with TFG (15 g/day for 8 weeks, n = 24) resulted in a significant reduction in FBG, ALT, and ALP levels compared to a placebo (n = 24). Also, compared to baseline, the levels of AST decreased significantly in the intervention group [190].

More recently, however, Chehregosha et al. (2024) failed to detect significant changes in glycemic indices, the lipid profile, or the prooxidant/antioxidant balance in patients with T2D (n = 23) following supplementation with TFG (335 mg of TFG dry seed extract three times daily for 8 weeks). TFG only improved HDL-c levels in the intervention group (n = 20) compared to baseline and the placebo group [191].

Finally, a 2024 SRMA of 19 RCTs (n = 1612) involving patients with T2D investigating the efficacy of TFG supplementation showed that TFG led to significant improvements in FBG, HbA_1c_, HOMA-IR, BMI, TC, LDL-c, and HDL-c concentrations. No effects were observed on FI, TG level, or BW in the overall analysis. Consistent heterogeneity was reported among the included clinical trials. Dosages of TFG (extract or powder) ranged from 25 to 50,000 mg/day, while the duration of interventions varied from 4to 24 weeks. In addition, TFG’s natural compounds may seem to act as powerful anti-inflammatory and antioxidant molecules to alleviate insulin signaling, but these findings are primarily based on preclinical studies [184].

*Clinical evidence in MASLD.* Evidence of the effect of TFG supplementation on liver function and histology in human subjects is limited (Table 10). A sole triple-blind controlled pilot clinical trial investigating the effect of TFG supplementation on the liver in patients with NAFLD was performed by Babaei et al. Thirty patients with NAFLD were randomly assigned to two groups, receiving either hydroalcoholic extract TFG (1 g/day for 3 months) (n = 13) or a placebo (n = 11). At baseline, all patients were given lifestyle advice. At the end of the study, some improvements in liver steatosis percentage and CAP score were observed among TFG-treated patients, but these changes were not statistically significant compared to those in the placebo group. In addition, no significant differences in ALT and AST levels were detected between the two groups. Anthropometric indices (BMI, BW, WHR) remained unchanged in both groups [192].

*Safety*. TFG supplementation is generally well tolerated. The most commonly reported adverse effects include nausea, diarrhea, dyspepsia, and flatulence, affecting up to 20% of study participants. Some subjects may also experience allergic reactions, probably due to TFG’s high protein antigen content. In addition, coumarin compounds in TFG have raised concerns regarding possible hepatotoxicity and anticoagulant effects; however, there is currently no evidence from clinical trials indicating these risks at standard therapeutic dosages [184].

*Summary.* To date, clinical research findings indicate that TFG has some beneficial effects on glycemic control and lipid profile in patients with T2D, but the study outcomes are inconsequential. The limited availability of RCTs investigating TFG’s therapeutic value in MASLD highlights the importance of further large, high-quality studies to confirm its potential clinical efficacy.

### 4.10. Ginger

Ginger (GGR) is derived from the rhizomes of the plant *Zingiber officinale* Roscoe and has been utilized for its antitumor, immunomodulatory, and antiemetic properties. The main functional compound of GGR is gingerol, which has been acknowledged as a potent anti-inflammatory molecule [193]. Other non-volatile components of GGR include shogaols, zingerones, and paradols [194]. According to clinical studies, ginger also exhibits hypoglycemic, hypolipidemic [194], and hepatoprotective activity [195].

*Clinical evidence in T2D.* Several RCTs have investigated the effects of GGR on glycemic control (Table 11). The first clinical study was performed in 2013 by Mahluji et al. and involved 58 patients with T2D who were administered GGR (n = 28) or a placebo (n = 30) for 2 months. The results showed that GGR supplementation led to significantly improved levels of HOMA-IR, QUICKI, insulin, LDL-c, and TGs relative to the placebo. However, no significant changes were observed concerning FBG, HbA_1c_, TC, and HDL-c concentrations or regarding BW [194].

Significantly improved glycemic control was reported by Khosravi et al. (2014) among patients with T2D treated with hypoglycemic agents and either GGR (n = 40) or placebo (n = 41) for 8 weeks. During the intervention, all patients were encouraged to maintain their usual diet and perform no physical activities. At the end of the study, GGR-supplemented patients exhibited a significant reduction in FBG and HbA_1c_ levels, as well as a significantly greater increase in the QUICKI index compared to the placebo group. HOMA-IR decreased significantly in both groups, but no significant differences between the groups were observed. On the other hand, BMI did not differ significantly between the groups either before or after the intervention [196].

Consistent with these findings, another 2014 RCT showed that, compared to placebo (n = 30), GGR (1600 mg) administered daily for 12 weeks was effective in improving several glucolipid and inflammatory markers in patients with T2D (n = 33). Study participants were asked to maintain their lifestyle habits during the trial. Significant decreases in plasma glucose, HbA_1c,_ HOMA-IR, insulin, TC, TGs, and CRP levels were detected following GGR supplementation. There was no significant difference between groups regarding TNF-α concentration. BW and BMI remained unchanged in both groups [197].

Similar results were reported following GGR supplementation with 3 g of GGR for 3 months in 45 patients with T2D. After the intervention, a significant decrease in serum glucose, HbA_1c_, insulin, HOMA-IR, and hs-CRP was observed in the GGR-treated participants. Also, they exhibited increased TAC and reduced MDA levels compared to the control group [198].

Makhdoomi Arzati et al. also confirmed GGR’s hypoglycemic effect in a 2017 RCT involving patients with T2D. GGR intake significantly reduced FBG and HBA_1c_ levels after 10 weeks compared to placebo. The ratio of LDL-c/HDL-c also decreased after GGR supplementation, although no other changes in the blood lipid profile were reported [199].

In 2020, Nunes Carvalho et al. demonstrated that GGR consumption improved both FBG and TC levels in T2D. The study participants were supplemented with either GGR (n = 47) or a placebo (n = 56) for 90 days. HbA_1c_ concentration declined more substantially in the intervention group compared to the control group, although this change was not statistically significant. Notably, LDL-c levels exhibited a greater reduction in the placebo control group [200].

A 2022 SRMA including 10 RCTs (n = 597) concluded that GGR consumption was effective in alleviating FBG and HbA_1c_ levels in patients with T2D. Nevertheless, in patients older than 50 years, no significant effect of GGR supplementation on HbA_1c_ levels was found. In addition, GGR intake failed to improve the blood lipid profile. GGR dosages ranged from 1200 to 3000 mg/day, whereas study durations varied from 8 to 13 weeks [203].

*Clinical evidence in MASLD.* RCTs investigating GGR’s effects in MASLD are scarce (Table 11). In 2016, Rahimlou et al. conducted a clinical trial involving 44 patients with NAFLD who were supplemented with GGR (2 g/day) or a placebo for 12 weeks. All study participants received lifestyle recommendations. Compared to the placebo, GGR intake was superior in improving ALT, GGT, HOMA-IR, hs-CRP, and TNF-α levels. Also, significant changes compared to the placebo were reported regarding liver steatosis grade. Nevertheless, GGR did not significantly affect AST levels or fibrosis score. Anthropometric parameters reduced significantly in both groups, but only the reduction in hip circumference was significantly different between the intervention and control groups [201].

Rafie et al. (2020) found that GGR (1500 mg/day) intake for 12 weeks led to a significant reduction in ALT, FBG, HOMA-IR, TC, LDL-c, and hs-CRP levels compared to placebo in patients with NAFLD. No significant differences between the two groups were observed concerning GGT, AST, FLI score, fatty liver grade, FI, HDL-c, TGs, TNF-α, APN, TAC, or BW. All patients followed a diet and physical activity regimen during the clinical trial [195].

Finally, a 2024 RCT reported that GGR supplementation may alleviate some metabolic dysfunction characteristics. Seventy-two patients with T2D and NAFLD were administered either GGR (2000 mg/day) or a placebo for 3 months. At the end of the study, no significant effects on liver steatosis and fibrosis scores were observed. Liver enzyme levels (AST, ALT, GGT) and anthropometric indices (BW, BMI, WC) decreased in both groups, without significant differences between them. HOMA-IR, HDL-c, and serum insulin levels improved compared to baseline in the GGR-treated patients [202].

*Safety*. GGR is recognized as safe by the Food and Drug Administration and has good tolerability with minimal side effects [204]. Reported adverse effects across RCTs include transient heartburn [201,202], dyspepsia, and nausea [202].

*Summary*. GGR consumption demonstrates promising potential as an adjuvant in T2D and MASLD management. Nevertheless, despite its hypoglycemic effects, GGR does not appear to significantly influence the blood lipid profile in T2D. In addition, GGR’s role in MASLD, particularly in T2D with MASLD, requires more in-depth investigation.

### 4.11. Soluble Fibers

Dietary fibers have long been recognized for their role in managing metabolic dysfunction. However, since modest improvements in glycemic control require unrealistic intake levels of dietary fibers (>50 g/zi), many fiber supplements have been developed, with extensive research highlighting their efficacy and ease of use [205]. Viscous, gel-forming fibers, such as psyllium, inulin-type fructans (ITFs), or guar gum, are among the most studied fibers that may be effective in alleviating metabolic dysfunction in patients with T2D [205,206,207] and MASLD [208] (Table 12).

Soluble fibers are often regarded as prebiotics due to their ability to promote the growth of beneficial gut microbial communities while reducing harmful bacteria [209]. The prebiotic effect of soluble fiber on GM-related outcomes in patients with T2D and NAFLD will be discussed in the next subsection.

*Clinical evidence in T2D.* A 2009 RCT evaluated the effect of psyllium (10.5 g/day) compared to placebo on glycemic control and lipid profiles in 40 patients with T2D treated with sulfonylureas and diet. Patients in both groups exhibited significantly lower FBG, HbA_1c_, BMI, and WC values after 2 months compared to baseline. However, TG concentration significantly decreased in the psyllium-treated group, but not in the control group (diet alone). The postprandial lipoprotein profile remained unchanged between the two groups [210].

*Psyllium.* Significant improvements in glucose metabolism parameters were reported by Feinglos et al. in a 2013 RCT assessing the dose–response effects of psyllium on metabolic control in 37 patients with T2D receiving conventional treatment. The patients were supplemented with either 6.8 or 13.6 g of psyllium/day or a placebo for 12 weeks. Both psyllium doses led to significantly lower FBG at weeks 4, 8, and 12. Moreover, HbA_1c_ levels significantly decreased compared to placebo at week 8 following supplementation with 13.6 g of psyllium and at week 12 with both dosages [211].

A 2015 SRMA of studies involving individuals with euglycemia, dysglycemia, and T2D concluded that in patients with T2D, psyllium was beneficial in significantly reducing the levels of FBG and HbA_1c._ Psyllium’s glycemic benefits were proportional to the baseline value of FBG, with no significant effects in normoglycemia, modest effects in prediabetes, and significant effects in T2D [212].

More recently, Abutair et al. (2018) also reported that compared to the control group (regular diet, n = 18), psyllium supplementation (10.5 g/day for 8 weeks) led to significant reductions in FBG, TC, TGs, and WC in newly diagnosed patients with T2D (regular diet, n = 18). The LDL-c level declined in both groups, but not significantly [213].

Psyllium has also been shown to have some beneficial effects on improving inflammation status. As such, a 2018 open RCT showed that patients with T2D following a moderate carbohydrate diet supplemented with psyllium (n = 20) exhibited significantly reduced fasting TNF-α and FI levels compared to baseline and to individuals receiving a low-carbohydrate diet and placebo (n = 17). It should be noted that these changes took place irrespective of weight changes since no significant variations in BW were reported between the two groups. No significant changes within groups or differences between groups were observed regarding FBG, PBG, and postprandial insulin. Postprandial TNF-α concentrations were significantly reduced compared to baseline in the intervention group, but there was no significant difference when compared to placebo controls [214].

**Table 12:** Summary of RCTs investigating the hypoglycemic, hypolipidemic, and anti-steatosis effects of soluble fibers.

Disease	Participants (Total)	Intervention	Control	Duration	Main Outcomes */**	References
T2D	n = 40	Psyllium 10.5 g/day + diet	Diet	2 months	↓ FBG, HbA_1c_, BMI, WC **	[210]
T2D	n = 37	Psyllium 6.8 or 13.6 g/day	Placebo	12 weeks	↓ HbA_1c_ *	[211]
T2D	n = 36	Psyllium 10.5 g/day	Regular diet	8 weeks	↓ FBG, TC, TGs, WC *	[213]
T2D	n = 37	Psyllium 7 g/day	Placebo	2 weeks	↓ fasting TNF-α, FI *	[214]
T2D	n = 49	Inulin 10 g/day	Placebo	2 months	↓ FBG, HbA_1c_, BMI, BW, MDA, ↑ TAC, SOD *	[215]
T2D	n = 49	Inulin 10 g/day	Placebo	8 weeks	↓ FBG, HbA_1c_, HOMA-IR, FI, hs-CRP, TNF-α *	[216]
T2D	n = 52	Inulin 10 g/day	Placebo	8 weeks	↓ FBG, HbA_1c_, IL-6, TNF-α *	[217]
T2D	n = 49	Inulin (chicory) 10 g/day	Placebo	2 months	↓ FBG, HbA_1c_, ALP *	[218]
T2D	n = 44	PHGG 10 g/day	Placebo	6 weeks	↓ HbA_1c,_ WC **	[219]
T2D	n = 79	Whey protein 17 g GG 5 g	Flavored placebo	12 weeks	↓ PBG, HbA_1c_ *	[220]
NASH	n = 7	Inulin 16 g/day	Placebo	8 weeks	↓ AST *	[221]
NAFLD	n = 75	Psyllium 10 g/day +LSI	Ground wheat 10 g/day + LSI	10 weeks	↓ ALT, BMI, BW, BF, WC *	[222]
NAFLD	n = 70	PP 10 g/day OB 10 g/day PP + OB 10 g/day	Placebo	12 weeks	no changes	[223]

* Statistically significant between groups (*p* < 0.05); ** Statistically significant from baseline (*p* < 0.05); “↓”, decreased; “↑”, increased. Abbreviations: ALT, alanine aminotransferase; ALP, alkaline phosphatase; AST, aspartate aminotransferase; BMI, body mass index; BF, body fat; BW, body weight; FBG, fasting blood glucose; CRP, C reactive protein; GG, guar gum; FI, fasting insulin; HbA_1c,_ glycosylated hemoglobin; HOMA-IR, homeostatic model assessment of insulin resistance; IL-6, interleukin 6; LSI, lifestyle intervention; MDA, malondialdehyde; OB, *Ocimum basilicum;* PBG, postprandial blood glucose; PHGG, partially hydrolyzed guar gum; PP, *Plantago psyllium*; SOD, superoxide dismutase; TC, total cholesterol; TAC, total antioxidant capacity; TGs, triglycerides; TNF-α, tumor necrosis factor α; WC, waist circumference.

Finally, a 2024 SRMA of 19 RCTs (n = 962) revealed that, compared to placebo, psyllium significantly decreased FBG, HbA_1c_, and HOMA-IR, but not serum insulin. Psyllium’s effect on FBG was effective at dosages both below and above 10 g/day with interventions lasting longer than 50 days. No significant changes in HbA_1c_ were observed with psyllium intake below 10 g/day. HOMA-IR and insulin levels showed no significant differences with psyllium consumption either below or above 10 g/day [224].

*Inulin*. Three RCTs involving women with T2D and overweight (n = 49, n = 49, n = 52) showed that supplementation with 10 g of inulin or oligofructose-enriched inulin versus placebo for 8 weeks was effective in significantly [215,216,217] improving glycemic control, anthropometric indices, oxidant and inflammation status. As such, Gargari et al. observed significant changes in FBG, HbA_1c_, MDA, TAC, and SOD after inulin consumption (n = 24) compared to the placebo (n = 25). No significant difference was observed regarding HOMA-IR between the two groups. Both BMI and BW decreased significantly in the inulin-treated patients relative to placebo controls after 2 months [215]. In another RCT, between-group comparisons showed a significant decline in FBG, HbA_1c_, HOMA-IR, FI, hs-CRP, and TNF-α in the inulin group (n = 24) compared to the placebo group (n = 25). IL-10 also increased in the inulin-treated patients, but this change was not significant. BW and BMI significantly decreased after 2 months in the intervention group, but not in the control group [216]. Finally, the third study reported significant improvements in FBG, HbA_1c_, IL-6, and TNF-α after inulin supplementation (n = 27) compared to the placebo (n = 25). Changes in hs-CRP and IL-10 levels were also noted in the intervention group, but these changes were not statistically significant. At the end of the study, both BMI and BW declined significantly after inulin consumption, while remaining unchanged following placebo intake [217].

A 2015 SRMA by Liu et al. involving healthy individuals, individuals with DLD, and those with overweight/obesity and T2D (n = 607) reported that ITF supplementation may decrease LDL-c levels in all studied populations. In addition, according to the T2D subgroup analysis (three RCTs), inulin supplementation was positively correlated with decreased FBG and FI levels, as well as increased HDL-c concentration [207].

In 2016, Farhangi et al. tested the effects of enriched chicory inulin supplementation (10 g/day) versus placebo on glucose parameters and liver enzymes in female patients with T2D (n = 49). After 2 months, significant reductions in FBG and HbA_1c_ were reported in the intervention group (n = 27) compared to baseline and placebo recipients (n = 22). AST levels also decreased significantly compared to baseline in the chicory-treated patients, while ALP concentration declined significantly in the intervention group compared to both baseline and controls. No significant changes were observed in ALT levels [218].

Finally, Wang et al. (2019) conducted an SRMA involving 33 RCTs (n = 1346) in healthy individuals and patients with obesity or T2D supplemented with ITFs. The results showed that overall, prebiotics were effective in reducing FBG, HbA_1c_, FI, and HOMA-IR levels, particularly in individuals with prediabetes and T2D. Glycemic control improved following supplementation of ≥10 g of ITFs/day for 6 weeks or longer [225].

*Partially hydrolyzed guar gum.* Dall’Alba et al. (2013) investigated the effects of supplementation with partially hydrolyzed guar gum (PHGG, 10 g/day for 6 weeks) versus placebo on cardiovascular risk factors in patients with T2D and MS (n = 44). PHGG-treated patients (n = 23) experienced a significant reduction in HbA_1c_ levels and WC after 4 and 6 weeks compared to baseline. No changes were observed in FBG, the blood lipid profile, or CRP in either group [219].

A 12-week trial by Watson et al. (2019) reported that supplementation with 150 mL of a flavored drink containing either 17 g of whey protein and 5 g of guar gum (n = 37) versus a placebo (n = 42), taken 15 min before breakfast and dinner daily, led to a significant reduction in gastric emptying and PBG compared to a placebo. The rate of gastric emptying was correlated with the degree of blood glucose decrease. Also, a modest but significant decline in HbA_1c_ levels was observed at the end of the study in the interventional group compared to the control group. There were no differences in BW between the two groups [220].

Pooled data from 14 RCTs included in a 2023 SRMA showed that guar gum is effective in reducing HbA_1c_ levels, without influencing FBG concentrations or BW. However, according to a subgroup analysis, an intake above 15 g/day of guar gum was shown to significantly improve FBG levels in patients with T2D [226]. Guar gum was also reported to significantly reduce TC and LDL-c concentrations in patients with T2D at a dosage ranging from 5 to 30 g/day. TG levels also decreased following supplementation of ≥ 20 g of guar gum/day. No effect on HDL-c concentration was observed [206].

*Clinical evidence in MASLD.* An overview of the RCTs discussed in this subsection is provided in Table 12. In 2005, a pilot study on the effects of soluble fiber in patients with NASH (n = 7) was performed by Daubioul et al. After 8 weeks of supplementation with 16 g of inulin/day, a significant decrease in plasma levels of AST was observed, but not in ALT, ALP, or GGT. No changes were reported in glucose and lipid metabolism parameters [221].

A 2015 trial involving patients with NAFLD (n = 75) who were supplemented with either 10 g of psyllium (n = 38) or ground wheat (n = 37) daily for 10 weeks, in addition to diet and increased physical activity, reported that fiber intake led to a significant reduction in BMI, WC, BF%, and serum ALT levels. The reduction in AST was not significant [222].

A four-arm, parallel, single-blind RCT (2016) compared the impact of psyllium *(Plantago psyllium*, PP) versus basil (*Ocimum basilicum*, OB) supplementation on anthropometric indices in patients with NAFLD (n = 70). Participants were allocated to four groups, each receiving one of the following supplementations: 10 g of PP/day; 10 g of OB/day; a combination of psyllium and OB at 10 g/day; or a placebo. After 12 weeks, the results failed to show any significant differences in BMI, BW, or body composition-related parameters between the intervention and control groups. The authors argued that the ineffectiveness of PP and OB supplementation could be explained by the low dosage of fibers administered [223].

However, a 2020 SRMA (n = 242) investigating the metabolic outcomes in NAFLD patients following soluble fiber intake reported that supplementation at a dosage of 10–16 g/day for 10 to 12 weeks results in favorable effects on anthropometric (BMI), metabolic (HOMA-IR, FI), and liver-related parameters (ALT, AST) [208].

*Safety*. Soluble fibers have proven efficient and are well tolerated in the management of CMDs. However, excessive intake may cause gastrointestinal symptoms, such as abdominal cramps, flatulence, constipation, or diarrhea [227].

*Summary*. Soluble fiber supplements may represent a viable and safe strategy to improve glycemic control, lipid profile, and hepatic steatosis in patients with T2D and MASLD. In addition, soluble fibers may have beneficial effects on anthropometric parameters, which also help enhance the metabolic health outcomes of these patients.

### 4.12. Omega-3 Fatty Acids

Omega-3 fatty acids (ω-3 PUFA) of marine origin are polyunsaturated fatty acids that exist in two forms: eicosapentaenoic acid (EPA) and docosahexaenoic acid (DHA) [228]. Research suggests that ω-3 PUFA exert cardioprotective benefits, although their effects on the plasma lipid profile are rather modest [229]. ω-3 PUFA are also associated with hypoglycemic activity [228], anti-steatosis effects [230], and body composition improvements [229].

*Clinical evidence in T2D*. An in-depth meta-analysis regarding the effects of ω-3 PUFA in T2D (n = 1533) was published in 2009. Hartweg et al. reported that compared to placebo, long-term supplementation with marine-derived ω-3 PUFA was effective in reducing TG levels, highlighting a potential dose–response effect. TG levels decreased with the administration of higher doses of ω-3 PUFA (>2 g/day). Also, a small but significant increase in LDL-c concentration was observed following supplementation. Other cardioprotective effects associated with ω-3 PUFA, such as attenuated fibrinogen and platelet aggregation, were also reported in this review [231].

In 2012, Crochemore et al. performed a single-blind trial involving 41 women with T2D who were given 2.5 g of fish oil/day (n = 14), 1.5 g of fish oil/day (n = 14), or a placebo (n = 13) for 30 days (Table 13). Study participants were encouraged to maintain their lifestyle habits throughout the trial. Patients receiving the lower dose of ω-3 PUFA experienced a greater decrease in BW and WC than patients supplemented with a higher dose. Also, among patients taking the lower dose of ω-3 PUFA, more frequent reductions in FBG, HbA_1c_, and TC levels were observed, as well as an increase in HDL-c concentration. Moreover, the higher dose of ω-3 PUFA showed a tendency to reduce insulin sensitivity. Since the lower dose of ω-3 PUFA was more effective in improving the study’s outcomes, the authors concluded that ω-3 PUFA supplementation is not essential for women with T2D treated with oral hypoglycemics to improve BW, body composition, or the lipid profile [229].

Short-term ω-3 PUFA supplementation may enhance the anti-inflammatory status of patients with T2D. In a 2012 RCT, Malekshahi et al. (2012) demonstrated that patients supplemented with 2.7 g of ω-3 PUFA/day (n = 42) exhibited significantly different levels of interleukin 2 and TNF-α after 8 weeks compared to placebo recipients (n = 42). No changes were observed in CRP concentration at the end of the study, which is likely the result of a small sample size [232].

In a 2016 double-blind RCT, 166 patients with T2D were randomized to receive either fish oil (2 g of ω-3 PUFA/day, n = 58), flaxseed oil (2.5 g of alpha-linolenic acid/day, n = 53), or corn oil (control, n = 65) for 180 days. Supplementation with ω-3 PUFA led to significant improvements in HbA_1c,_ TC, TC/HDL, and TG levels compared to the control group. No changes were observed regarding FBG, HOMA-IR, or FI. Flaxseed oil intake did not significantly influence any of the abovementioned parameters [233].

Wang et al. (2017) administered either 2.4 g/day of ω-3 PUFA or a placebo (corn oil) to 99 patients with T2D. After 6 months, significant improvements in TGs and HDL-c levels were observed in the ω-3 PUFA group (n = 49) compared to the placebo group (n = 50), although FBG, HbA_1c_, HOMA-IR, insulin, TC, and LDL-c levels remained unchanged [234].

ω-3 PUFA anti-inflammatory activity was also investigated by Jacobo-Cejudo et al. (2017) in a RCT involving 54 patients with T2D. All study participants maintained a regular diet and medication during the 24-week trial. Results showed that glucose, TGs, and WC declined significantly only in the ω-3 PUFA group, while HbA_1c_ decreased significantly in both groups at the end of the study. In contrast, HOMA-IR and insulin levels increased significantly in all study participants. No significant changes were observed in other anthropometric indices (BMI, BW, WHR, BF%) or APN levels in either group [235]. In 2018, O’Mahoney et al. conducted the largest meta-analysis and meta-regression assessing the impact of ω-3 PUFA supplementation in T2D. This review, involving 45 RCTs (n = 2674), showed that ω-3 PUFA intake was associated with significant reductions in HbA_1c_, LDL, and TG levels. In addition, ω-3 PUFA exhibited anti-inflammatory effects by decreasing both TNF-α and IL-6 concentrations. However, other markers related to glycemic control, lipid profile, and inflammatory status did not change following supplementation. The doses of ω-3 PUFA ranged from 0.4 to 18 g, while the duration of the intervention varied from 2 to 104 weeks [242].

Finally, a 2023 RCT involving patients with T2D treated with glimepiride led to significant improvements in FBG, HbA_1c,_ HOMA-IR, TC, TGs, LDL-c, and HDL-c levels following 12 weeks of ω-3 PUFA consumption (n = 35) compared to a placebo (n = 35) [236].

*Clinical evidence in MASLD.* Numerous RCTs and several SRMAs have evaluated the effects of ω-3 PUFA supplementation on liver function in patients with NAFLD (Table 13). In 2008, Spadaro et al. performed a RCT involving 36 patients with NAFLD who were treated with either 2 g of ω-3 PUFA/day and an American Heart Association (AHA) recommended diet (n = 18) or an AHA regular diet (n = 18). After 6 months, both groups experienced significant weight loss. In addition, significant decreases in levels of ALT, GGT, TGs, HOMA-IR, and TNF-α were observed in the ω-3 PUFA group, but not in the diet group. HDL concentration also improved after ω-3 PUFA intake. Full reversal of steatosis was observed in over one-third of the supplemented patients, while half of them exhibited an overall decrease in the severity of NAFLD. However, no complete regression of hepatic fat accumulation was noted with diet alone, although some improvements in the steatosis score occurred in almost 30% of participants [237].

In a 2012 SRMA of nine studies (n = 355), Parker et al. reported beneficial effects on liver fat and AST levels following ω-3 PUFA supplementation. ALT levels were not significantly decreased. Notably, when RCT data were considered, only the efficacy of ω-3 PUFA on liver fat content remained significant. The median dose of ω-3 PUFA was 4 g/day (0.8–13.7 g/day), and the median duration of treatment was 6 months (8 weeks–12 months) [243].

Seventy patients with NAFLD associated with hyperlipidemia were randomly allocated to consume ω-3 PUFA (n = 36, 4 g/d) or a placebo (n = 34) for 3 months in a 2015 double-blind RCT. ω-3 PUFA led to significant reductions in the levels of glucose, TC, TGs, ApoB, and the liver enzymes ALT and GGT compared to placebo recipients. Significant improvements in inflammation-related markers, specifically APN, TNF-α, and CK18-M30, were also observed after ω-3 PUFA supplementation. No significant differences between groups were noted regarding anthropometric indices, HOMA-IR, LDL, HDL-c, and hs-CRP levels [238].

A phase 2 RCT conducted in 2014 assessed the efficacy of ethyl-eicosapentanoic acid (EPA-E), a synthetic PUFA, in patients with NASH and significant NAFLD (n = 243). Study participants were administered a low dose of EPA-E (1800 mg/day), a high dose of EPA-E (2700 mg/day), or a placebo for 12 months. Results failed to show any significant improvements in NASH-related histopathology, liver enzymes, glycemic control, BW, hs-CRP, or APN levels. Nevertheless, TG concentrations were significantly reduced compared to the placebo group following high-dose EPA-E treatment [239].

In line with these results, Dasarathy et al. (2015) demonstrated that a daily intake of 3.6 g of ω-3 PUFA for 48 weeks led to no significant changes in BW, body composition, or liver enzyme levels in patients with well-controlled T2D and NASH. Moreover, at the end of the study period, ω-3 PUFA worsened glucose control and histological parameters compared to the placebo [240].

However, in their 2016 SRMA of seven RCTs involving 442 patients with NAFLD/NASH, He et al. showed that ω-3 PUFA supplementation is effective in improving the lipid profile, particularly TGs, but also TC and HDL-c levels, as well as ALT concentration. The doses of ω-3 PUFA used in the included trials varied between 0.83 and 6.4 g/day, and the duration of the studies ranged from 6 to 18 months [244].

Finally, one year of ω-3 PUFA treatment (3.6 g/day) led to a significant decrease in GGT levels in 30 patients with NAFLD and MS compared to placebo recipients (n = 30). Moreover, ω-3 PUFA seemed to promote liver fat reduction in patients who also experienced weight loss. According to the study’s results, a significant association between BW decrease and liver fat reduction was observed, but only among the ω-3 PUFA-treated patients. However, BW or HFC did not change significantly in either group at the end of the intervention. Similarly, no changes in noninvasive NASH-related parameters or liver fibrosis scores were observed [241].

*Safety*. ω-3 PUFA intake has an excellent safety profile, with minimal to no long-term side effects, particularly following supplementation with a dose below the highest approved. Both the U.S. Food and Drug Administration and the EFSA consider ω-3 PUFA supplementation safe at doses up to 5 g/day [228]. However, doses greater than 3 g/day are likely to increase LDL-c levels, particularly in patients with hypertriglyceridemia [245].

*Summary*. Clinical evidence suggests that ω-3 PUFA may be an effective therapeutic adjuvant in T2D, as they exhibit a modest yet beneficial influence on glucose and lipid parameters. In MASLD, improvements in liver steatosis-related parameters and lipid profiles, particularly TGs, were also reported following ω-3 PUFA supplementation. However, its effectiveness in alleviating MASH features remains uncertain.

## 5. NBCs for GM Modulation in T2D and MASLD

A reduced Bacteroidetes-to-Firmicutes ratio has long been proposed as a marker for dysbiosis in individuals with obesity. However, this ratio may not adequately reflect the metabolic dysfunction in patients with T2D, since its increase was positively associated with reduced glucose tolerance [11,246]. The GM profile of patients with T2D exhibits a higher abundance of pathogenic and opportunistic Gram-negative microbes, including *Enterobacteriaceae*, *Clostridiales*, *E. coli*, *Bacteroides caccae*, *Prevotella copri*, and *Bacteroides vulgates*. Gram-negative bacteria (e.g., Bacteroidetes) are associated with high levels of LPS [11]. Moreover, several genera have been reported to have a consistent negative correlation with T2D, specifically *Bifidobacterium*, *Bacteroides*, *Faecalibacterium*, *Roseburia,* and *Akkermansia*, while *Ruminococcus*, *Fusobacterium*, and *Blautia* have been found to be increased in patients with T2D [246].

Compared to healthy individuals, patients with MASLD display increased levels of Proteobacteria, Enterobacteriaceae, *Escherichia,* and *Dorea* and decreased levels of Rikenellaceae, Ruminococaceae, *Faecalibacterium*, *Eubacterium*, and *Prevotella* [247]. Notably, there is a partial overlap between the MASLD-related GM profile and the GM dysbiotic signature described in T2D. For example, Proteobacteria has a higher prevalence in both T2D and MASLD patients [11,247], whereas low levels of *Faecalibacterium prausnitzii* are also reported in patients with T2D or obesity [248]. In addition, *Bacteroides vulgates*, which is reportedly increased in T2D, severe obesity, and IR, has also been shown to be abundant in patients with liver fibrosis [248].

GM acts as a key modulator of the host’s physiology, partly through certain metabolites, such as SCFAs and LPS [58]. Altered levels of SCFAs (acetate, propionate, and butyrate) are pivotal in the onset and development of CMDs. For instance, a reduction in butyrate-producing species (*Roseburia intestinalis*, *Faecalibacterium prausnitzii*) is involved in the pathogenesis of T2D [11]. Elevated levels of LPS, a major outer wall component of Gram-negative bacteria, result in metabolic endotoxemia, triggering a state of low-grade inflammation and IR [58]. In the setting of endotoxemia, LPS promotes liver lipogenesis and augments intrahepatic inflammation [249].

LPS-binding protein (LPB) is an acute-phase protein that forms a complex with LPS and facilitates its binding to cellular receptors (TLR4/CD14), with the subsequent initiation of inflammatory signaling pathways. As such, LBP is involved in the regulation of immune responses by modulating inflammatory reactions induced by LPS. Whereas the role of LPS in the development of CMDs is well-known, the effects of LPB are not clear [249]. Some studies have reported increased circulating levels of LPB in obesity and obesity-related diseases, including T2D and MASLD [58,250,251], while others have not confirmed these findings [249].

### Prebiotics

**Figure 3:**
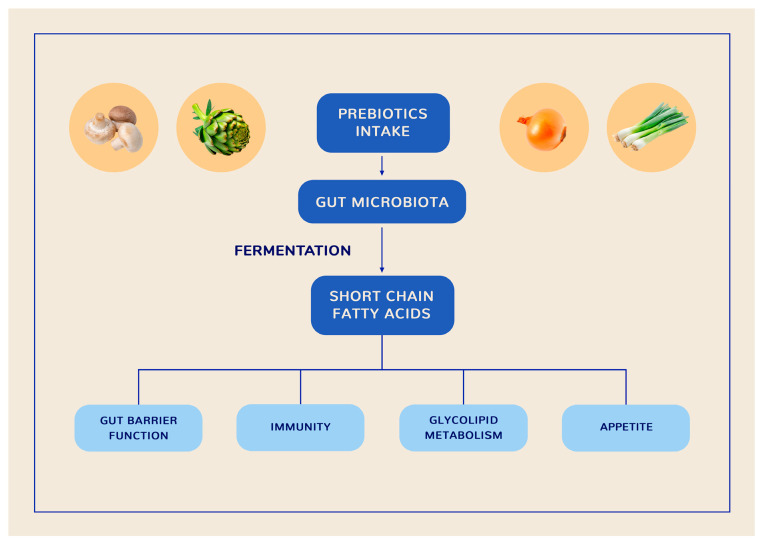
Impact of prebiotics on metabolic function via SCFAs [252].

Prebiotics are nondigestible carbohydrates, polyunsaturated fatty acids, or polyphenols that can be converted to SCFAs [11]. SCFAs influence metabolic health by reducing IR, curbing appetite, decreasing lipolysis, and enhancing energy expenditure (Figure 3) [252].

The carbohydrate prebiotics include inulin, fructooligosaccharides (FOS), and galactooligosaccharides (GOS). Inulin and FOS are collectively known as ITFs. Prebiotics beneficially influence GM by increasing the number of *Lactobacillus* and/or *Bifidobacterium* species [253]. Indeed, studies on healthy adults have shown an increased bifidogenic effect at doses as low as 5 g of ITFs/day, with an optimal dose of 10 g/day [254]. However, in T2D, there is a complex interplay between prebiotics and GM, which is influenced not only by the dysbiosis that accompanies this condition but also by the effects of antidiabetic medication. Metformin, for instance, has antibiotic-like properties, altering GM independently of prebiotic intake and consequently confounding the results of clinical trials assessing the composition of gut bacteria in T2D [253]. Currently, only a limited number of interventional trials in T2D and MASLD have included GM-specific outcome measures following prebiotic supplementation (Table 14).

A 2014 study involving female patients with T2D demonstrated that compared to placebo, inulin supplementation (10 g/day for 8 weeks, n = 24) was effective in improving glycemic control and reducing plasma LPS levels [216]. In a second study by the same authors involving 52 women with T2D, 8 weeks of prebiotic supplementation (10 g/day of oligofructose-enriched inulin, n = 27) led to significant reductions in glucose metabolism parameters and inflammatory markers, including LPS, compared to placebo recipients [217].

Pedersen et al. (2016) conducted a RCT investigating the relationship among intestinal permeability, glucose control, and intestinal bacteria in men with well-controlled T2D (n = 29). Compared to a placebo, supplementation with a mixture of GOS (5.5 g/day for 12 weeks) had no significant effects on glucose tolerance or bacterial populations. Nevertheless, prebiotic-treated patients displayed a significant increase in microbial diversity and richness indices after the intervention. In addition, an inverse relationship between changes in *Veillonellaceae* and changes in both glucose response and IL-6 levels was reported. The authors argued that the lack of significant shifts in GM composition could be attributed to metformin use and high variability in T2D characteristics among the study participants [255].

Another RCT was performed by Gonai et al. (2017) involving 52 patients with T2D who were given either 10 g/day of GOS syrup (n = 27) or 10 g/day of a placebo (n = 25) for 1 month. The baseline assessment revealed that compared to healthy controls (n = 25), *Veillonellaceae* was significantly more abundant in the T2D group and was positively correlated with BMI, FBG, HbA_1c_, TGs, and LBP. It was proposed that *Veillonellaceae* might play a role in the onset of glucose intolerance. In contrast, the abundance of *Bifidobacteriaceae*, *Clostridiales*, *Incertae sedis XIV*, and *Peptostreptococcaceae* were significantly lower in patients with T2D. Moreover, T2D patients also exhibited significantly lower microbial diversity compared to controls. However, while LPS were not detected in any study participant, LBP was significantly higher in patients with T2D and was also correlated with FBG and HbA_1c_ levels. No significant differences regarding IL-6, IL-10, and TNF-α were observed between T2D patients and healthy controls. Results showed that GOS supplementation led to a significant restoration of *Bifidobacteriaceae* abundance in patients with T2D but had no significant effect on LPB or glucose tolerance. Also, the levels of *Peptostreptococcaceae*, *Ruminocaccaceae*, *Lachnospiraceae*, *Porphyromonadaceae*, and *Erysipelotrichaceae* significantly decreased compared to baseline after GOS intake. Both inflammatory and anti-inflammatory markers remained unchanged [58].

In 2017, Canfora et al. investigated the effect of supplementation with GOS (15 g/day for 12 weeks) versus a placebo on the GM composition and metabolic parameters of 44 individuals with excess weight and prediabetes. GOS supplementation led to a five-fold increase in the abundance of *Bifidobacterium* species. However, microbial richness and diversity were not significantly affected by prebiotic intake compared to the placebo group. In addition, no significant differences between the two groups were reported regarding the levels of fecal or plasma SCFAs or concentrations of incretins, gut hormones, insulin sensitivity, anthropometric indices, LBP, or other inflammatory markers (IL-6, IL-8, TNF-α) [256].

Birkeland et al. (2020) reported in a crossover RCT that 6-week supplementation with ITFs (16 g/day) led to a significant increase in bifidobacteria (*B. adolescentis*) and SCFAs in the feces of patients with T2D (n = 25) compared to a placebo. No effects on butyric acid or overall microbial diversity were observed [254].

A 2022 SRMA conducted by Ojo et al. revealed that, in patients with T2D, prebiotic supplementation led to a significant reduction in HbA_1c_ levels compared to the controls. Prebiotic intake was also associated with an increase in the relative abundance of beneficial bacteria, including *Bifidobacterium* and *Akkermansia*, although this increase was not statistically significant compared to the control group. Metformin, on the other hand, decreased *Bifidobacterium* but increased *Lactobacillus* and *Akkermansia*; however, these changes were not significantly different compared to those in the control groups. Neither prebiotics nor oral antidiabetic medication exerted significant effects on BMI, FBG, or PBG compared to the controls [252].

Finally, in a 2023 12-week double-blind placebo-controlled RCT, 192 participants with T2D were assigned to receive either a prebiotic fiber-rich supplement (n = 95), a placebo fiber-absent supplement (n = 48), or dietary advice alone (n = 49). All study participants were asked to follow nutritional recommendations. The fiber-based supplement featured a mixture of resistant starch (RS) and oat beta-glucan, which may attenuate postprandial glycemic response. Pivotal bacterial species involved in RS degradation to enhance butyrate-producing species include *Ruminococcus bromii* and *Bifidobacterium* ssp. (primary degraders) and *Clostridia* clusters IV and XIVa (secondary degraders). The results showed no significant changes in microbial composition (alpha diversity) at the end of the study in either group. Compared to the diet-managed patients, the relative abundance of RS primary degraders increased in the prebiotic-treated patients, but not significantly. Instead, there was a significant increase in the relative abundance of secondary degraders, specifically butyrate-producing bacteria (*Roseburia faecis* and *Anaerostipes hadrus*) after prebiotic intake. Also, HbA_1c_ levels and BW were significantly reduced after supplementation in the prebiotic-treated group versus the placebo group. Since FBG levels showed no significant changes either within or between groups after 12 weeks, and weight loss was rather modest, the authors suggested that glycemic control was improved, probably due to attenuation of PBG and other factors [257].

Only two studies specifically investigated the effects of prebiotic supplementation on GM in patients with NAFLD/NASH (Table 14). In a 2019 pilot clinical trial, Bomhof et al. demonstrated that the administration of oligofructose (8 g/day for 12 weeks followed by 16 g/day for 24 weeks, n = 8) compared to a placebo (n = 6) led to improved liver steatosis and an improved overall NAS score, independently of weight loss, in patients with NASH. However, no changes in liver enzyme levels (ALT, ALP, GGT) or glycemic indices were observed after the intervention. IL-6 and TNF-α concentrations decreased after prebiotic consumption, but not significantly compared to the placebo group. Oligofructose has also been shown to enhance *Bifidobacterium* and decrease microorganisms within *Clostridium* clusters XI and I. While the beneficial bifidogenic effect is well-documented, the implications of reduced bacteria from *Clostridium* clusters XI and I in NASH are not clear [258].

Reshef et al. (2024) investigated the effect of prebiotic supplementation on liver function, fecal GM, metabolism, and inflammation in patients with NAFLD and MS. All study participants were advised to follow a weight-maintenance diet. Patients supplemented with 16 g of ITFs/day for 12 weeks (n = 8) exhibited a significant increase in *Bifidobacterium* compared to placebo recipients (n = 11). However, no other significant changes were observed regarding hepatic (liver enzymes—ALT, AST, GGT—or HFC), metabolic (FBG, HbA_1c_, HOMA-IR, insulin, TC, TGs, LDL-c, HDL-c), or inflammatory markers (CRP) between the two groups. Notably, patients’ weight did not fluctuate throughout the course of the study. The authors concluded that prebiotics, in the absence of weight loss, may not alleviate NAFLD-related outcomes [259].

*Safety*. There are no safety-related concerns regarding the intake of ITFs and GOS [260,261]. ITFs are well tolerated in dosages up to 20 g/day [260], while the recommended dose for GOS ranges from 8 to 15 g/day. Higher intakes may lead to gastrointestinal discomfort, flatulence, diarrhea, or cramping [261].

*Summary*. So far, prebiotics possess great potential as an approach to ameliorate metabolic dysfunction in T2D and MASLD via GM modulation. However, although evidence regarding their beneficial health effects is accumulating, there is still insufficient clinical data, and the results of available studies are less conclusive.

## 6. Conclusions

NBCs have sparked significant interest in recent decades, with evidence highlighting their role in improving glycemic control, lipid profile, and liver function by providing powerful antioxidant and anti-inflammatory benefits. Our findings show that certain NBCs, particularly BBR, CRM, soluble fibers, and omega-3 fatty acids, have substantial evidence regarding their efficacy and safety in the management of T2D and MASLD. The ability of NBCs to counteract metabolic dysfunction positions them as a promising adjunct therapy to conventional treatment, as well as candidates for new drug development. Future well-designed investigations are needed to deepen our understanding of NBCs’ specific action mechanisms, determine optimal dosage and intervention duration, and optimize their therapeutic potential in T2D and MASLD.

## Figures and Tables

**Table 1:** Cardiometabolic risk factors for MASLD diagnosis [14].

Cardiometabolic Criteria
BMI ≥ 25 kg/m^2^ or WC > 94 cm (male) or 80 cm (female)
Fasting serum glucose ≥ 100 mg/dL or 2 h post-load glucose levels ≥ 140 mg/dL or HbA_1c_ ≥ 5.7% or T2D or T2D medication
Blood pressure ≥ 130/85 or hypertension medication
Plasma triglycerides ≥ 150 mg/dL or lipid-lowering medication
Plasma HDL-c ≤ 40 mg/dL (male) or ≤50 mg/dL (female) or lipid-lowering medication

Abbreviations: BMI = body mass index; WC = waist circumference; HbA_1c_ = glycosylated hemoglobin; HDL-c = high-density lipoprotein cholesterol.

**Table 2:** Summary of RCTs investigating the hypoglycemic, hypolipidemic, and anti-steatosis effects of BBR.

Disease	Participants (Total)	Intervention	Control	Duration	Main Outcomes */**	References
T2D	Study A: n = 31 Study B: n = 43	BBR 1500 mg/day BBR 1500 mg/day + T2D treatment	Metformin 1500 mg/day -	3 months	Study A: ↓ FBG, PBG, HbA_1c_ **, TGs, TC * Study B: ↓ FBG, PBG, HbA_1c_, HOMA-IR, FI, TC, LDL-c **	[68]
T2D	n = 97	BBR 1 g/day	Metformin 1.5 g/day RSG 4 mg/day	2 months	↓ FBG, HbA_1c_, TGs **	[69]
T2D	n = 60	BBR 1 g/day	Placebo	3 months	↓ FBG, PBG, HbA_1c_, TGs, TC, LDL-c *	[70]
T2D	n = 63	BBR + SLM (1000/210 mg/day)	BBR 1000 mg/day	4 months	↓ HbA_1c_ *	[71]
T2D	n = 45	BBR + SLM + statins BBR + SLM + ezetimibe	BBR + SLM (1000/210 mg/day)	12 months	↓ FBG, HbA_1c_, TC, LDL-c **	[72]
T2D	n = 69	BBR 300 mg/day	Standard treatment	24 months	↓ CRP, MDA, ↑ HMW-APN, GSH-Px, SOD, TAC *	[73]
NAFLD	n = 155	BBR 1.5 g/day + LSI	LSI	16 weeks	↓ HFC, BW, HOMA-IR, TC, TGs *	[74]
NAFLD	n = 80	BBR 1.5 g/day + LSI	LSI	16 weeks	↓ HFC, TC, TGs, BMI, BW, WC *	[75]
NASH/T2D	n = 88	BUDCA 2000 mg/day	Placebo	18 weeks	↓ HFC, HbA_1c_, ALT, GGT, BW *	[76]
MAFLD	n = 63	BBR 1500 mg/day	Placebo	12 weeks	↓ ALT, AST/ALT ratio, TC *	[77]

* Statistically significant between groups (*p* < 0.05); ** Statistically significant from baseline (*p* < 0.05); “↓”, decreased; “↑”, increased. Abbreviations: ALT, alanine aminotransferase; AST, aspartate aminotransferase; BBR, berberine; BW, body weight; BUDCA, berberine ursodeoxycholate; CRP, C-reactive protein; FBG, fasting blood glucose; GGT, gamma-glutamyl transpeptidase; GSH-Px, glutathione peroxidase; HbA_1c,_ glycosylated hemoglobin; HOMA-IR, homeostatic model assessment of insulin resistance; HFC, hepatic fat content; HMW-APN, high molecular weight adiponectin; LDL-c, low-density lipoprotein cholesterol; LSI, lifestyle intervention; PBG, postprandial blood glucose; SLM, silymarin; SOD, superoxide dismutase; TC, total cholesterol; TAC, total antioxidant capacity; TGs, triglycerides; WC, waist circumference.

**Table 3:** Summary of RCTs investigating the hypoglycemic, hypolipidemic, and anti-steatosis effects of CRM.

Disease	Participants (Total)	Intervention	Control	Duration	Main Outcomes */**	References
T2D	n = 100	CRMs 300 mg/day	Placebo	3 months	↓ FBG, HbA_1c_, HOMA-IR, TGs *	[85]
T2D	n = 70	CRM 80 mg/day	Placebo	3 months	↓ FBG, HbA_1c,_ BMI *	[86]
T2D	n = 100	CRM 1000 mg/day + piperine 10 mg/day	Placebo	12 weeks	↓ TC, ↑ HDL-c *	[83]
T2D	n = 95	nano-CRM + EPA	Placebo	12 weeks	↓ insulin, hs-CRP, ↑ TAC *	[84]
T2D	n = 229	CRM 1500 mg/day	Placebo	12 months	↓ FBG, HbA_1c_, HOMA-IR, ↑ APN *	[87]
T2D	n = 227	CRM 1500 mg/day	Placebo	12 months	↓ LDL-c, ApoB, hs-CRP, IL-6, TNF-α *	[88]
NAFLD	n = 77	CRM 500 mg/day	Placebo	8 weeks	↓ HFC, ALT, AST, FBG, HbA_1c,_ TC, TGs, LDL-c *	[89]
NAFLD	n = 48	CRM 1500 mg/day	Placebo	12 weeks	↓ ALT, AST, hs-CRP, TNF-α, BMI, BW, WC, steatosis/fibrosis **	[90]
NAFLD	n = 50	CRM 1500 mg/day + LSI	Placebo + LSI	12 weeks	↓ FLI, FLS **	[91]
NAFLD	n = 60	CRM 500 mg/day + piperine 5 mg/day	Placebo	12 weeks	↓ ALT, AST, TC, LDL-c, FBG, WC *	[92]

* Statistically significant between groups (*p* < 0.05); ** Statistically significant from baseline (*p* < 0.05); “↓”, decreased; “↑”, increased. Abbreviations: ALT, alanine aminotransferase; APN, adiponectin; ApoB, apolipoprotein B; AST, aspartate aminotransferase; BMI, body mass index; BW, body weight; FBG, fasting blood glucose; FLI, fatty liver index; FLS, fatty liver score; HbA_1c,_ glycosylated hemoglobin; HDL-c, high-density lipoprotein cholesterol; HOMA-IR, homeostatic model assessment of insulin resistance; hs-CRP, high-sensitivity C-reactive protein; HFC, hepatic fat content; IL-6, interleukin-6; LDL-c, low-density lipoprotein cholesterol; LSI, lifestyle intervention; TC, total cholesterol; TGs, triglycerides; TNF-α, tumor necrosis factor α; WC, waist circumference.

**Table 4:** Summary of RCTs investigating the hypoglycemic, hypolipidemic, and anti-steatosis effects of RSV.

Disease	Participants (Total)	Intervention	Control	Duration	Main Outcomes */**	References
T2D	n = 57	RSV 250 mg/day	T2D medication	3 months	↓ HbA_1c_, TC **	[97]
T2D	n = 14	RSV 1000 mg/day	Placebo	5 weeks	no significant changes	[98]
T2D	n = 17	RSV 150 mg/day	Placebo	30 days	no significant changes	[99]
T2D	n = 179	RSV 500 mg or 40 mg/day	Placebo	6 months	no significant changes	[100]
T2D + CHD	n = 56	RSV 500 mg/day	Placebo	4 weeks	↓ FBG, HOMA-IR, ↑ HDL-c, QUICKI, TAC, ↓ MDA **	[101]
T2D	n = 110	RSV 200 mg/day	Placebo	24 weeks	FBG, HbA_1c_, HOMA-IR, FI, hs-CRP, TNF-α, IL-6 **	[102]
NAFLD	n = 20	RSV 3000 mg/day	Placebo	8 weeks	no significant changes	[103]
NAFLD	n = 50	RSV 500 mg + LSI	Placebo	12 weeks	↓ ALT *, AST BMI **	[104]
NAFLD	n = 60	RSV 600 mg/day	Placebo	3 months	↓ ALT, AST, glucose, HOMA-IR, TC, LDL-c, TNF-α, CK18-M30, ↑ APN *	[105]
NAFLD	n = 26	RSV 1500 mg/day	Placebo	6 months	no significant changes	[106]

* Statistically significant between groups (*p* < 0.05); ** Statistically significant from baseline (*p* < 0.05). “↓”, decreased; “↑”, increased. Abbreviations: ALT, alanine aminotransferase; APN, adiponectin; AST, aspartate aminotransferase; BMI, body mass index; CK18-M30, cytokeratin 18 M30; FBG, fasting blood glucose; FI, fasting insulin; HbA_1c,_ glycosylated hemoglobin; HDL-c, high-density lipoprotein cholesterol; HOMA-IR, homeostatic model assessment of insulin resistance; hs-CRP, high-sensitivity C-reactive protein; IL-6, interleukin-6; LDL-c, low-density lipoprotein cholesterol; MDA, malondyladehyde; QUICKI, quantitative insulin sensitivity check index; TAC, total antioxidant capacity; TC, total cholesterol TNF-α, tumor necrosis factor.

**Table 5:** Summary of RCTs investigating the hypoglycemic, hypolipidemic, and anti-steatosis effects of ACNs.

Disease	Participants (Total)	Intervention	Control	Duration	Main Outcomes */**	References
T2D	n = 37	ACNs 350 mg every 8 h	Placebo	2 months	↓ FBG, 2h-PBG, HbA_1c_ *	[112]
T2D	n = 58	ACNs 320 mg/day	Placebo	24 weeks	↓ FBG, HOMA-IR, TGs, LDL-c *, ↑ HDL-c, APN *	[113]
Prediabetes/T2D	n = 138	ACNs 320 mg/day	Placebo	12 weeks	↓ FBG, ↑ APN * (only in TD2)	[114]
T2D	n = 52	22 g freeze-dried blueberries	Placebo	8 weeks	↓ HbA_1c_, TGs, AST, ALT *	[115]
T2D	n = 20	1.4 g bilberry extract/day	Placebo	4 weeks	No significant changes	[116]
Prediabetes/T2D	n = 40	ACNs 320 mg/day	-	4 weeks	↓ IL-6, TNF-α *	[117]
NAFLD	n = 36	HS extract 2700 mg/day	Placebo	12 weeks	↓ WC, WHR, BF, FFA *	[118]
NAFLD	n = 74	ACNs 320 mg/day	Placebo	12 weeks	↓ ALT, CK18-M30, MPD *	[119]
NAFLD	n = 40	CMFE 20 mL/day	Placebo	12 weeks	No significant changes	[120]
MAFLD	n = 108	CMFP 30 g/day + diet	Diet	8 weeks	↓ AST, ALT, GGT, FBG, HbA_1c_, HOMA-IR, TC, TGs, LDL-c, BW, BF, WC, CRP **	[121]

* Statistically significant between groups (*p* < 0.05); ** Statistically significant from baseline (*p* < 0.05); “↓”, decreased; “↑”, increased. Abbreviations: ACNs, anthocyanins; ALT, alanine aminotransferase; APN, adiponectin; AST, aspartate aminotransferase; BF, body fat; BW, body weight; CMFE, *Cornus mas* L. fruit extract; CMFP, *Cornus mas* L. fruit powder; CK18-M30, cytokeratin 18 M30; CRP, C-reactive protein; FFA, free fatty acids; FBG, fasting blood glucose; HbA_1c,_ glycosylated hemoglobin; GGT, gamma-glutamyl transpeptidase; HDL-c, high-density lipoprotein cholesterol; HOMA-IR, homeostatic model assessment of insulin resistance; HS, *Hibiscus sabdariffa*; IL-6, interleukin-6; LDL-c, low-density lipoprotein cholesterol; MPD, myeloperoxidase; 2-h PBG, 2-h postprandial blood glucose; TC, total cholesterol; TGs, triglycerides; TNF-α, tumor necrosis factor; WC, waist circumference; WHR, waist-to-hip ratio.

**Table 7:** Summary of RCTs investigating the hypoglycemic, hypolipidemic, and anti-steatosis effects of phenolic compounds and carotenoids.

Disease	Participants (Total)	Intervention	Control	Duration	Main Outcomes */**	References
T2D	n = 72	ALE 1200 mg/day	Placebo	2 months	↓ TC, LDL-c *	[145]
MS	n = 68	ALE 1800 mg/day	Placebo	12 weeks	↓ TGs *	[140]
T2D	n = 52	Tomato juice 500 mL/day	Placebo	4 weeks	↑ resistance to LDL oxidation **	[146]
T2D	n = 64	HES 500 mg/day	Placebo	6 weeks	↓ TNF-α, IL-6, hs-CRP *	[147]
MS	n = 49	HES 1000 mg/day + LSI	Placebo	12 weeks	↓ FBG, TGs, TNF-α *	[148]
NASH	n = 60	AE 2700 mg/day	Placebo	2 months	↓ ALT, AST, TC, TGs *	[149]
NAFLD	n = 90	ALE 600 mg/daily	Placebo	2 months	↓ ALT, AST, TC, TGs, LDL-c, HDL-c *	[143]
T2D/ NAFLD	n = 80	Cyc + BPF 300 mg/day	Placebo	16 weeks	↓ ALT, AST, GGT, ALP, TNF-α *	[150]
NAFLD	n = 49	HES 1 g/day + LSI	Placebo	12 weeks	↓ ALT, GGT, TC, TGs, hs-CRP, TNF-α *	[151]
NAFLD	n = 92	HES 1 g/day + LSI Flaxseeds 30 g/day + LSI HES + flaxseeds + LSI	LSI	12 weeks	↓ FBG, HOMA-IR, ALT, FLI *	[152]

* Statistically significant between groups (*p* < 0.05); ** Statistically significant from baseline (*p* < 0.05); “↓”, decreased; “↑”, increased. Abbreviations: ALE, artichoke leaf extract; ALT, alanine aminotransferase; ALP, alkaline phosphatase; AST, aspartate aminotransferase; BPF, bergamot phenolic fraction; Cyc, *Cynara cardunculus*; FLI, fatty liver index; FBG, fasting blood glucose; GGT, gamma-glutamyl transpeptidase; HES, hesperidin; HDL-c, high-density lipoprotein cholesterol; HOMA-IR, homeostatic model assessment of insulin resistance; hs-CRP, high-sensitivity C-reactive protein; IL-6, interleukin-6; LDL-c, low-density lipoprotein cholesterol; LSI, lifestyle intervention; TC, total cholesterol; TGs, triglycerides; TNF-α, tumor necrosis factor α.

**Table 8:** Summary of RCTs investigating the hypoglycemic, hypolipidemic, and anti-steatosis effects of SLM.

Disease	Participants (Total)	Intervention	Control	Duration	Main Outcomes */**	References
T2D	n = 51	SLM 600 mg/day	Placebo	4 months	↓ FBG, HbA_1c_, TC, TGs, LDL-c, ALT, AST *	[161]
T2D	n = 40	SLM 420 mg/day	Placebo	45 days	↓ hs-CRP, ↑ GPx, SOD, TAC *	[162]
T2D	n = 40	SLM 420 mg/day	Placebo	45 days	↓ FBG, SI, HOMA-IR, TGs, ↑ QUICKI, HDL-c *	[163]
T2D	n = 60	SLM 140 mg/kg/day SLM + AT AT	Placebo	8 weeks	↓ FBG, SI, HOMA-IR, ALT, AST, ALP *	[164]
T2D	n = 48	SLM 420 mg/day + diet	Diet	12 weeks	no changes	[165]
NAFLD	n = 36	SLM 1080.6 mg/day + Vitamin E 36 mg/day + LSI	LSI	3 months	↓ GGT, BW, WC **	[166]
NAFLD	n = 150	SLM 140 mg/day Metformin 500 mg/day Pioglitazone 15 mg/day Vitamin E 400 UI/day	Placebo + LSI	3 months	↓ ALT, AST, TGs, LDL-c **	[167]
NASH	n = 99	SLM 2100 mg/day + LSI	Placebo + LSI	48 weeks	↓ TGs *	[168]
MASLD	n = 83	SLM 103.2 mg/day	Placebo	24 weeks	↓ GGT, LSM, ApoB *	[169]

* Statistically significant between groups (*p* < 0.05); ** Statistically significant from baseline (*p* < 0.05); “↓”, decreased; “↑”, increased. Abbreviations: ALT, alanine aminotransferase; ALP, alkaline phosphatase; ApoB, apolipoprotein B; AST, aspartate aminotransferase; AT, aerobic training; BW, body weight; FBG, fasting blood glucose; GGT, gamma-glutamyl transpeptidase; GPx, glutathione peroxidase; HDL-c, high-density lipoprotein cholesterol; HbA_1c,_ glycosylated hemoglobin; HOMA-IR, homeostatic model assessment of insulin resistance; hs-CRP, high-sensitivity C-reactive protein; LDL-c, low-density lipoprotein cholesterol; LSI, lifestyle intervention; LSM, liver stiffness measurement; QUICKI, quantitative insulin sensitivity check index; SI, serum insulin; SLM, silymarin; SOD, superoxide dismutase; TC, total cholesterol; TAC, total antioxidant capacity; TGs, triglycerides; WC, waist circumference.

**Table 9:** Summary of RCTs investigating the hypoglycemic, hypolipidemic, and anti-steatosis effects of CNM.

Disease	Participants (Total)	Intervention	Control	Duration	Main Outcomes */**	References
T2D	n = 60	CNM 1, 3 or 6 g/day	Placebo	40 days	↓ glucose, TC, TGs **	[174]
T2D	n = 58	CNM 2 g/day	Placebo	12 weeks	↓ HbA_1c_ *	[175]
T2D	n = 66	CNM 120 mg/day or 360 mg/day	Placebo	3 months	↓ FBG, HbA_1c_ **	[176]
T2D	n = 37	CNM 3 g/day	Placebo	8 weeks	↓ FBG, HbA_1c_, TGs, BW, BMI, BF **	[177]
T2D	n = 138	CNM 1 g/day	Placebo	3 months	↓ FBG, 2-h PBG, HOMA-IR, FI, TC, LDL-c, ↑ HDL-c	[178]
T2D	n = 39	CNM 3 g/day	Placebo	8 weeks	no significant changes	[179]
NAFLD	n = 45	CNM 1.5 g/day +LSI	Placebo + LSI	12 weeks	↓ ALT, AST, GGT, TC, TGs, FBG, HOMA-IR, QUICKI, hs-CRP *	[180]

* Statistically significant between groups (*p* < 0.05); ** Statistically significant from baseline (*p* < 0.05); “↓”, decreased; “↑”, increased. Abbreviations: ALT, alanine aminotransferase; AST, aspartate aminotransferase; BF, body fat; BMI, body mass index; BW, body weight; CNM, cinnamon; FBG, fasting blood glucose; FI, fasting insulin; GGT, gamma-glutamyl transpeptidase; HDL-c, high-density lipoprotein cholesterol; HbA_1c,_ glycosylated hemoglobin; HOMA-IR, homeostatic model assessment of insulin resistance; hs-CRP, high-sensitivity C-reactive protein; LDL-c, low-density lipoprotein cholesterol; LSI, lifestyle intervention; QUICKI, quantitative insulin sensitivity check index; 2-h PBG, 2-h postprandial blood glucose; TC, total cholesterol; TGs, triglycerides.

**Table 10:** Summary of RCTs investigating the hypoglycemic, hypolipidemic, and anti-steatosis effects of TFG.

Disease	Participants (Total)	Intervention	Control	Duration	Main Outcomes */**	References
T2D	n = 25	TFG extract 1 g/day	Placebo + LSI	2 months	↓ HOMA-IR, TGs, ↑ HDL-c *	[185]
T2D	n = 69	TFG extract 6.3 g/day + LSI	Placebo + LSI	12 weeks	↓ FBG, 2-h PBG, HbA_1c_ *	[186]
T2D	n = 88	TFG seeds 10 g/day	Placebo	8 weeks	↓ FBG, HbA_1c_, SI, HOMA-IR, TC, TGs, ↑ APN *	[187]
T2D	n = 9	TFG 2 g/day + Metformin	Glibenclamide 5 mg/day + Metformin	12 weeks	↑ SI, HDL/LDL ratio **	[188]
T2D	n = 95	TFG seed powder solution 50 g/day	Metformin	1 month	↓ TC, TGs, HDL-c, LDL-c *	[189]
T2D	n = 48	TFG seed powder 15 g/day + LSI	LSI	8 weeks	↓ FBG, ALT, ALP *	[190]
T2D	n = 43	TFG extract 1005 mg/day	Placebo	8 weeks	↑ HDL-c *	[191]
NAFLD	n = 24	TFG extract 1 g/day + LSI	Placebo + LSI	3 months	no changes	[192]

* Statistically significant between groups (*p* < 0.05); ** Statistically significant from baseline (*p* < 0.05); “↓”, decreased; “↑”, increased. Abbreviations: ALT, alanine aminotransferase; ALP, alkaline, phosphatase; APN, adiponectin; FBG, fasting blood glucose; HDL-c, high-density lipoprotein cholesterol; HbA_1c,_ glycosylated hemoglobin; HOMA-IR, homeostatic model assessment of insulin resistance; LDL-c, low-density lipoprotein cholesterol; LSI, lifestyle intervention; 2-h PBG, 2-h postprandial blood glucose; SI, serum insulin; TC, total cholesterol; TGs, triglycerides.

**Table 11:** Summary of RCTs investigating the hypoglycemic, hypolipidemic, and anti-steatosis effects of GGR.

Disease	Participants (Total)	Intervention	Control	Duration	Main Outcomes */**	References
T2D	n = 58	GGR 2 g/day	Placebo	2 months	↓ HOMA-IR, insulin, LDL-c, TGs, ↑ QUICKI *	[194]
T2D	n = 81	GGR 3 g/day	Placebo	8 weeks	↓ FBG, HbA_1c,_ ↑ QUICKI *	[196]
T2D	n = 63	GGR 1600 mg/day	Placebo	12 weeks	↓ glucose, HbA_1c,_ HOMA-IR, insulin, TC, TGs, CRP *	[197]
T2D	n = 45	GGR 3 g/day	Placebo	3 months	↓ glucose, HbA_1c,_ insulin_,_ HOMA-IR, hs-CRP, MDA, ↑ TAC *	[198]
T2D	n = 45	GGR 2000 mg/day	Placebo	10 weeks	↓ FBG, HbA_1c_ *	[199]
T2D	n = 103	GGR 1.2 g/day	Placebo	90 days	↓ FBG, TC *	[200]
NAFLD	n = 44	GGR 2 g/day + LSI	Placebo	12 weeks	↓ ALT, GGT, CAP, HOMA-IR, hs-CRP, TNF-α *	[201]
NAFLD	n = 46	GGR 1500 mg/day + LSI	Placebo	12 weeks	↓ ALT, FBG, HOMA-IR, TC, LDL-c, hs-CRP *	[195]
T2D/NAFLD	n = 72	GGR 2000 mg/day	Placebo	3 months	↓ HOMA-IR, insulin, ↑ HDL-c **	[202]

* Statistically significant between groups (*p* < 0.05); ** Statistically significant from baseline (*p* < 0.05); “↓”, decreased; “↑”, increased. Abbreviations: ALT, alanine aminotransferase; CAP, controlled attenuation parameter score; CRP, C-reactive protein; FBG, fasting blood glucose; GGR, ginger; GGT, gamma-glutamyl transpeptidase; HDL-c, high-density lipoprotein cholesterol; HbA_1c,_ glycosylated hemoglobin; HOMA-IR, homeostatic model assessment of insulin resistance; hs-CRP, high-sensitivity C-reactive protein; LDL-c, low-density lipoprotein cholesterol; LSI, lifestyle intervention; MDA, malondialdehyde; QUICKI, quantitative insulin sensitivity check index; TAC, total antioxidant capacity; TC, total cholesterol; TGs, triglycerides; TNF-α, tumor necrosis factor.

**Table 13:** Summary of RCTs investigating the hypoglycemic, hypolipidemic, and anti-steatosis effects of ω-3 PUFA.

Disease	Participants (Total)	Intervention	Control	Duration	Main Outcomes */**	References
T2D	n = 41	ω-3 PUFA 2.5 g/ ω-3 PUFA 1.5 g	Placebo	30 days	↓ BW, WC *	[229]
T2D	n = 84	ω-3 PUFA 2.7 g/day	Placebo	8 weeks	↓ IL-2, TNF-α *	[232]
T2D	n = 166	ω-3 PUFA 2 g/day ALA 2.5 g/day	Placebo	180 days	↓ HbA_1c_, TC, TGs, TC/HDL, LDL-c *	[233]
T2D	n = 99	ω-3 PUFA 2.4 g	Placebo	6 months	↓ TGs, ↑ HDL-c *	[234]
T2D	n = 54	ω-3 PUFA 520 mg/day	Placebo	24 weeks	↓ serum glucose, WC, TGs **	[235]
T2D	n = 70	ω-3 PUFA 1 g/day	Placebo	12 weeks	↓ FBG, HbA_1c,_ HOMA-IR, TC, TGs, LDL-c,* ↑ HDL-c *	[236]
NAFLD	n = 36	ω-3 PUFA 2 g/day	Diet	6 months	↓ ALT, GGT, TGs, HOMA-IR, BMI, TNF-α **, ↑ HDL-c **	[237]
NAFLD	n = 70	ω-3 PUFA 4 g/day	Placebo	3 months	↓ glucose, TC, TGs, ApoB, ALT GGT, TNF-α, CK18-M30, ↑ APN *	[238]
NASH	n = 243	EPA-E 1800 mg/day 2700 mg/day	Placebo	12 months	↓ TGs *	[239]
NASH/T2D	n = 37	ω-3 PUFA 3.6 g + LSI	Placebo + LSI	48 weeks	↑ FBG, HOMA-IR **	[240]
NAFLD	n = 60	ω-3 PUFA 3.6 g + LSI	Placebo	1 year	↓ GGT *	[241]

* Statistically significant between groups (*p* < 0.05); ** Statistically significant from baseline (*p* < 0.05); “↓”, decreased; “↑”, increased. Abbreviations: ALA, alpha-linolenic acid; ALT, alanine aminotransferase; ApoB, apolipoprotein B; APN, adiponectin; BMI, body mass index; BW, body weight; CK18-M30, cytokeratin 18 M30; EPA-E, ethyl-eicosapentanoic acid; FBG, fasting blood glucose; GGT, gamma-glutamyl transpeptidase; HbA_1c,_ glycosylated hemoglobin; HDL-c, high-density lipoprotein cholesterol; HOMA-IR, homeostatic model assessment of insulin resistance; IL-2, interleukin 2; LDL-c, low-density lipoprotein cholesterol; LSI, lifestyle intervention; ω-3 PUFA, ω-3 poliunsaturated fatty acids; TC, total cholesterol; TGs, triglycerides; TNF-α, tumor necrosis factor α; WC, waist circumference.

**Table 14:** Summary of RCTs investigating the hypoglycemic, hypolipidemic, and anti-steatosis effects of prebiotics.

Disease	Participants (Total)	Intervention	Control	Duration	Main Outcomes */**	References
T2D	n = 49	Inulin 10 g/day	Placebo	8 weeks	↓ LPS *	[216,217]
T2D	n = 52	Inulin 10 g/day	Placebo	8 weeks	↓ LPS *	[217]
T2D	n = 29	GOS 5.5 g/day	Placebo	12 weeks	↑ bacterial diversity and richness **	[255]
T2D	n = 52	GOS 10 g/day	Placebo	4 weeks	↑ *Bifidobacteriaceae* *, ↓ *Peptostreptococcaceae*, *Ruminocaccaceae*, *Lachnospiraceae*, *Porphyromonadaceae*, *Erysipelotrichaceea* **	[58]
Prediabetes	n = 44	GOS 15 g/day	Placebo	12 weeks	↑ Bifidobacterium *	[256]
T2D	n = 25	ITFs 16 g/day	Placebo	6 weeks	↑ *Bifidobacterium*, SCFAs *	[254]
T2D	n = 192	RS, OBG + diet	Placebo Diet	12 weeks	↑ *Roseburia faecis*, *Anaerostipes hadrus*, ↓ HbA_1c_ *	[257]
NASH	n = 14	ITF 8 g/day ITF 16 g/day	Placebo	12 weeks 24 weeks	↑ *Bifidobacterium*, ↓ *Clostridium* clusters XI and I, steatosis *	[258]
NAFLD	n = 19	ITF 16 g/day	Placebo	12 weeks	↑ Bifidobacterium *	[259]

* Statistically significant between groups (*p* < 0.05); ** Statistically significant from baseline (*p* < 0.05); “↓”, decreased; “↑”, increased. Abbreviations: GOS, galactooligosaccharides; HbA_1c_, glycosylated hemoglobin; ITFs, inulin-type fructans; LPS, lipopolysaccharide; RS, resistant starch; OBG, oat beta-glucan; SCFAs, short-chain fatty acids.
